# Functional Ambidexterity of an Ancient Nucleic Acid‐Binding Domain

**DOI:** 10.1002/anie.202505188

**Published:** 2025-05-08

**Authors:** Orit Weil‐Ktorza, Segev Naveh‐Tassa, Yael Fridmann‐Sirkis, Dragana Despotović, Kesava Phaneendra Cherukuri, Tatsuya Corlett, Yaakov Levy, Norman Metanis, Liam M. Longo

**Affiliations:** ^1^ Institute of Chemistry The Center for Nanoscience and Nanotechnology Casali Center of Applied Chemistry The Hebrew University of Jerusalem Jerusalem 9190401 Israel; ^2^ Department of Chemical and Structural Biology Weizmann Institute of Science Rehovot 7610001 Israel; ^3^ Department of Life Sciences Core Facilities Weizmann Institute of Science Rehovot 7610001 Israel; ^4^ Department of Biomolecular Sciences Weizmann Institute of Science Rehovot 7610001 Israel; ^5^ Institute of Molecular Genetics and Genetic Engineering University of Belgrade Belgrade 11042 Serbia; ^6^ Institute of Science Tokyo Earth‐Life Science Institute Tokyo 152–8550 Japan; ^7^ Blue Marble Space Institute of Science Seattle Washington 98104 USA

**Keywords:** Chemical protein synthesis, Mirror‐image proteins, Nucleic acid‐binding proteins, Origin of life, Protein evolution

## Abstract

The helix‐hairpin‐helix (HhH) motif is an ancient and ubiquitous nucleic acid‐binding element that has emerged as a model system for studying the evolution of dsDNA‐binding domains from simple peptides that phase separate with RNA. We analyzed the entire putative evolutionary trajectory of the HhH motif – from a flexible peptide to a folded domain – for functional robustness to total chiral inversion. Against expectations, functional “ambidexterity” was observed for both the phase separation of HhH peptides with RNA and binding of the duplicated (HhH)_2_‐Fold to dsDNA. Moreover, dissociation kinetics, mutational analysis, and molecular dynamics simulations revealed an overlap between the binding modes adopted by the natural and mirror‐image proteins to natural dsDNA. The similarity of several dissociation phases upon chiral inversion may reflect the history of (HhH)_2_‐Fold binding, with the ultimate emergence of a high‐affinity binding mode, supported by a bridging metal ion, depopulating but not displacing more primitive (potentially ambidextrous) modes. These data underscore the surprising functional robustness of the HhH protein family and suggest that the veil between worlds with alternative chiral preferences may not be as impenetrable as is often assumed.

## Graphical abstract

The (HhH)_2_‐Fold is an ancient and ubiquitous nucleic acid‐binding domain. We demonstrate that this domain, which interacts with the minor groove of dsDNA, is functionally ambidextrous – that it can bind to dsDNA of either chirality. Moreover, binding of both chiral forms of dsDNA is mediated by an overlapping binding surface. These results reveal new insights into the evolution of the (HhH)_2_‐Fold and its mode of binding.

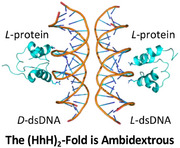

## Introduction

Life on Earth is characterized by exquisite homochirality: With few exceptions,^[^ ^1^ ^]^ proteins and peptides are composed of *L*‐amino acids (except for glycine, which is achiral) while nucleic acids (RNA and DNA) are derived from *D*‐ribose. The history of biological near‐homochirality is murky, no doubt because its origins predate the last universal common ancestor (LUCA), which likely arose more than 3.5 billion years ago.^[^ ^2^, ^3^, ^4^ ^]^ Consequently, theories regarding the emergence and evolution of homochirality remain speculative and tend to emphasize chemical and physical processes over biological selection.^[^ ^5^ ^]^ The benefits of chiral control over amino acid incorporation are, nevertheless, quite clear: by stabilizing and regularizing the conformations of proteins (especially protein secondary structures^[^ ^6^ ^]^), homochirality laid the groundwork for the emergence of stable, folded protein domains.

Whether *D*‐ribose and *L*‐amino acids are an optimal chiral pair in some fundamental sense is unknown. However, a recent report by Carell and co‐workers suggests a prebiotically plausible scenario for an RNA‐peptide world^[^ ^7^ ^]^ that would favor *L*‐homochirality in peptides given *D*‐ribose homochirality.^[^ ^8^ ^]^ Either way, total chiral inversion of a *contemporary* protein or substrate molecule typically results in a catastrophic loss of functionality, as demonstrated by Milton and Kent.^[^ ^9^ ^]^ Consequently, therapeutic compounds with inverted chirality are resistant to endogenous nucleases and proteases, conferring extended in vivo half‐lives,^[^ ^10^ ^]^ and mirror‐image enzymes deployed to degrade environmental pollution, such as achiral plastics, exhibit superior biostability.^[^ ^11^ ^]^ Indeed, these outcomes are sufficiently reliable that several technologies have been developed to leverage the special properties of mirror‐image biomolecules,^[^ ^12^ ^]^ including Spieglemers^[^ ^13^ ^]^ (aptamers with inverted chirality), mirror‐image phage display,^[^ ^14^, ^15^, ^16^, ^17^ ^]^ and most recently mirror‐image RaPID technology.^[^ ^18^, ^19^ ^]^ For some protein families, most notably chaperones,^[^ ^20^ ^]^ robustness to chiral inversion has been reported and is likely a consequence of the non‐specific nature of the mediating interactions. Thus, chiral inversion studies may provide a unique probe of the interplay between specific and non‐specific binding modes, particularly for interacting partners with multiple binding modes.

**Figure 1:**
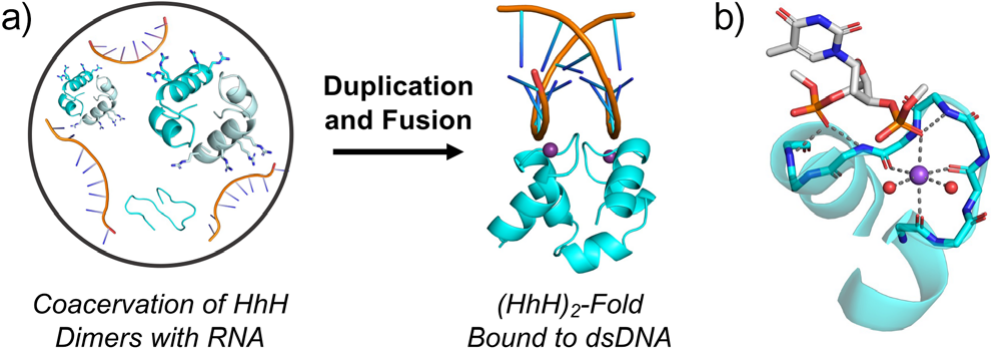
The HhH motif and the (HhH)_2_‐Fold. a) Transition from a dimerizing HhH motif that forms coacervates with RNA to a monomeric (HhH)_2_‐Fold that binds to the minor groove of dsDNA, a chiral surface. The unfolded monomer within the coacervate is meant to emphasize the complex folding and oligomerization dynamics within the droplets. The dsDNA‐bound Primordial‐(HhH)_2_ structure was predicted by AlphaFold3. b) The HhH motif binding mode (PDB ID 3i0x). Sequential phosphate groups are bound via the N‐terminus of an α‐helix as well as interactions mediated by a conserved sodium ion (*purple* sphere).^[^ ^33^ ^]^ Water molecules, drawn as *red* spheres, complete the octahedral geometry about the sodium ion. The segment **K**K**F** MG**V**GPQ (*cyan* sticks) contains the region of the conserved binding motif (underlined; in our constructs this region is PGIGP) and the residues that chelate the metal ion (bold). Structure figures were made using PyMOL.

We now report functional ambidexterity – a latent ability to interact with both natural and mirror‐image nucleic acids – along the entire putative evolutionary trajectory of an ancient and ubiquitous nucleic acid‐binding element. Our model protein is derived from the helix‐hairpin‐helix (HhH) motif,^[^ ^21^ ^]^ which was identified as among the primordial peptides around which modern domains condensed.^[^ ^22^ ^]^ Binding of the duplicated (HhH)_2_‐Fold^[^ ^23^ ^]^ to the minor groove of dsDNA (a symmetric, chiral surface) is not sequence specific and is associated with multiple kinetic phases. We have previously used the HhH motif to study the early functional evolution of nucleic acid binding, where we observed a transition from a flexible peptide that phase separates with RNA to a structured domain that binds to the minor groove of dsDNA (Figure 1a).^[^ ^24^ ^]^ More recently, we have demonstrated that dimerization and folding of the HhH motif, likely to form an (HhH)_2_‐Fold, is promoted upon RNA binding and occurs within peptide‐RNA coacervates.^[^ ^25^ ^]^

Mirror‐image forms of the (HhH)_2_‐Fold were chemically synthesized, and to our surprise, robustness to total chiral inversion was observed for both HhH peptide coacervation with RNA and (HhH)_2_‐Fold binding to dsDNA. With respect to dsDNA binding, we found that increasing the flexibility of the canonical binding loop and surrounding domain was significantly more disruptive to binding than total chiral inversion of the fold. Molecular dynamics simulations revealed that some of the residues that mediate binding of the *L*‐protein to *D*‐dsDNA (the natural pair) also participate in the *D*‐protein:*D*‐dsDNA (mirror protein:natural dsDNA) binding mode. An analysis of dissociation kinetics suggested the existence of multiple low‐affinity binding modes as well as a high‐affinity binding mode (or modes). The similarity between the dissociation kinetics of natural and unnatural binding pairs may indicate the existence of ambidextrous binding modes. Whether ancestors of the HhH motif and (HhH)_2_‐Fold ever bound *L*‐dsDNA in a natural setting – or ever will – is an intriguing (but unlikely) possibility. Instead, the apparent ambidexterity of the (HhH)_2_‐Fold is likely a manifestation of its functional versatility and may track the evolution of binding modes within this protein family.

## Results and Discussion

Proteins and peptides characterized in this study were prepared by chemical synthesis using solid‐phase peptide synthesis (SPPS)^[^ ^26^ ^]^ and native chemical ligation (NCL)^[^ ^27^ ^]^ followed by desulfurization of the Cys residue at the ligation site, as described previously^[^ ^24^, ^25^ ^]^ (see **SI** for a detailed protocol and Figures S1–S19 and Schemes S1 and S2). Construct names and sequences are given in Table 1.

**Table 1:** Peptide and protein sequences.^a)^

Construct name^a)^	Chiral form	Sequence^b)^
Precursor‐HhH	*L* & *D*	RIRRASVEELTEV PGIGP RLARRILERL
Precursor‐HhH	*D*/*L* ^c)^	rIrRaSvEeLtEv PGIGP rLaRrIlErL
Primordial‐(HhH)_2_	*L* & *D*	RIRRASVEELTEV PGIGP RLARRILERL *ASIE* RIRRASVEELTEV PGIGP RLARRILERL
Primordial‐(HhH)_2_‐5G	*L*	RIRRASVEELTEV GGGGG RLARRILERL *ASIE* RIRRASVEELTEV GGGGG RLARRILERL
Ancestor‐(HhH)_2_	*L*	HRKRRSKRTLRSELDDI PGIGP KTAKALLKHF *ASVE* KIKKASVEELTEV PGIGP KLAKKIYEHF

^a)^ Naming follows that of reference.^[^ ^24^ ^]^ However, since ornithine‐containing constructs were not tested in this study, the “‐Arg” signifier is dropped for simplicity.

^b)^ The location of the conserved PGIGP‐binding motif is underlined. Residues associated with the linker that joins two HhH motifs are italicized.

^c)^ Alternating *D*‐ and *L*‐amino acids. *D*‐amino acids are indicated with lowercase letters.

Circular dichroism (CD) spectra were collected for the constructs and contained 10 µM *L*‐Precursor‐HhH, *D*‐Precursor‐HhH, or *D/L*‐Precursor‐HhH in 5 mM Tris·HCl pH 7.5, 50 mM NaCl with either 0% or 20% (v/v) trifluoroethanol (TFE). Samples containing 5 µM *L*‐Primordial‐(HhH)_2_, *D*‐Primordial‐(HhH)_2_, or *L*‐Primordial‐(HhH)_2_–5G were measured in 5 mM Tris·HCl pH 7.5, 500 mM NaCl, 10 mM MgCl_2_, 5 mM CaCl_2_. All spectra were collected from 195 to 260 nm, and the spectrum of the buffer was subtracted (see **SI** for details).

For phase separation studies, peptides and polyuridylic acid (polyU) were dissolved in water. Coacervation was induced by mixing peptide, polyU, and MES pH 5.6. Droplets were observed using an inverted microscope (see **SI** for details).

Binding of 29‐bp *D*‐dsDNA and *L*‐dsDNA (mirror‐image DNA derived from *L*‐ribose; see Table S1 for DNA sequences) was measured by surface plasmon resonance (SPR). Since the (HhH)_2_‐Fold variants are positively charged at neutral pH, a C1 chip (S‐Series Cytiva, Sweden), which carries less negative charge than the standard CM5 chip, was used. Streptavidin was conjugated to the chip surface using EDC/NHS chemistry in the presence of acidified PBS buffer, as outlined in the C1 sensor chip manual. Approximately 2000 RU (Chip 1, buffer pH 3.8) or 700 RU (Chip 2, SI; buffer pH 4.6) of streptavidin was covalently conjugated to the chip surface and then the chip was blocked by injecting 1 M ethanolamine pH 8.0 for 5 min. Subsequently, 405 RU of *D*‐dsDNA and 423 RU of *L*‐dsDNA (Chip 1) or 95 RU of *D*‐dsDNA and 97 RU of *L*‐dsDNA (Chip 2) in which one strand of the duplex was 5′‐biotinulated was stably associated to the surface of one channel. See Table S1 for DNA sequences. Before data collection, a normalization cycle followed by three priming cycles were run to stabilize the instrument, and the reported sensorgrams were double subtracted: first, by the background binding of the analyte to a streptavidin‐conjugated control channel and then by the average of two buffer injection runs.

Protein–DNA interactions were analyzed by microscale thermophoresis (MST). Experiments were performed with 25 nM of synthetic, Cy5‐labelled (HhH)_2_‐Fold proteins, which were prepared by coupling Cy5‐NHS ester to the N‐terminus of synthetic proteins (note that there are no Lys residues present in these sequences), and the experiments were carried out at 25 °C (see **SI** for details). Labeled peptides were mixed with serially diluted dsDNA samples in 50 mM Tris pH 7.5, 150 mM NaCl, 0.05% Tween‐20 in premium capillaries. Laser power was set to 40%. Dissociation constants (*K*_D_) could not be calculated because binding is non‐specific for the minor groove of dsDNA and, as a result, each molecule of dsDNA has many degenerate, overlapping binding sites.

MD simulations were performed in GROMACS 2022^[^ ^28^ ^]^ with the CHARMM36 force field.^[^ ^29^ ^]^ Each system was solvated in a TIP3P water box with 0.125 M NaCl to ensure charge neutrality and then energy‐minimized and equilibrated under NVT and NPT ensembles.^[^ ^30^ ^]^ Production runs were performed at 300 K for 1 µs per replicate (three replicates per system) with a 2 fs timestep and a 1 nm cutoff for non‐bonded interactions. Structural models were generated using AlphaFold2,^[^ ^31^ ^]^ PyMOL (pymol.org), and Dstabilize,^[^ ^32^ ^]^ and positional restraints were applied to one end of the DNA molecule to mimic a longer linear molecule.

### The HhH Motif Binds Nucleic Acids via a Bridging Cation and the N‐terminus of an α‐helix

The conserved binding loop of the HhH motif interacts with two consecutive phosphate moieties of the phosphodiester backbone (Figure 1b). The 3′ phosphate is bound by the N‐terminal end of the binding loop and the 5′ phosphate of the next nucleotide is bound by the C‐terminal end of the binding loop. The binding modes of the two phosphate groups, however, are notably different: The phosphate moiety at the 5′ end sits atop the N‐terminus of an α‐helix. The other phosphate group interacts with both the protein backbone and a conserved cation, often sodium in crystal structures,^[^ ^33^ ^]^ that is situated in a nest formed by three backbone carbonyls (Figure S20). The geometry about the metal ion is nearly octahedral and often includes two water molecules in addition to the three backbone carbonyls and one oxygen donated by a nucleic acid phosphate group. Without a cation, AlphaFold3^[^ ^34^ ^]^ does not predict binding of Primordial‐(HhH)_2_ to dsDNA (Figure S20), further supporting its importance.^[^ ^33^ ^]^

### Coacervation of an HhH Motif with RNA Is Functionally Ambidextrous

Although coacervate formation with RNA can be achieved by simple peptides,^[^ ^35^ ^]^ we have previously demonstrated that the amino acid composition of the *L*‐Precursor‐HhH peptide (entry 1, Table 1) is not sufficient for droplet formation^[^ ^24^ ^]^: shuffling the sequence of *L*‐Precursor‐HhH – either completely or preserving the positions of the basic amino acids – resulted in a polypeptide that forms insoluble aggregates when mixed with *D*‐polyU.^[^ ^24^ ^]^ Subsequent studies demonstrated that binding to RNA and coacervation were associated with dimerization of the peptide.^[^ ^25^ ^]^

**Figure 2:**
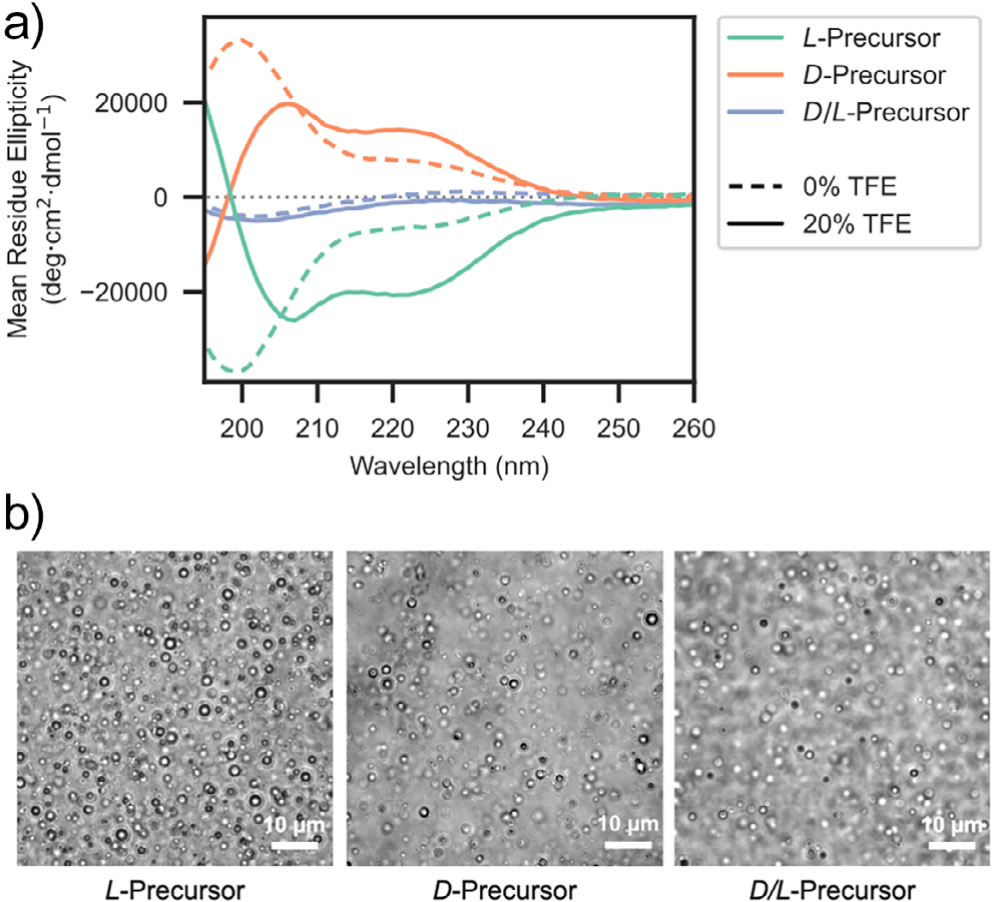
Coacervation of Precursor‐HhH with PolyU in response to chiral inversion. a) Circular dichroism spectra of *D*‐ and *L*‐ Precursor‐HhH peptides indicate that the peptides are largely unfolded and are mirror images of each other. Addition of trifluoroethanol (TFE) induces α‐helices in both cases, as indicated by the development of peaks at 208  and 222 nm. The *D*/*L*‐Precursor‐HhH peptide (entry 2, Table 1), which is composed of amino acids of alternating chirality, does not exhibit significant circular dichroism signal alone or in the presence of TFE. b) Light micrographs of coacervates formed by 300 µM peptide and 1 mg/mL PolyU. *D*/*L*‐Precursor‐HhH consistently exhibited reduced coacervate formation.

To explore the functional profile of Precursor‐HhH and its degree of ambidexterity in greater detail, *L*‐ and *D*‐Precursor‐HhH were chemically synthesized (Table 1; see **SI** for details). Note that all chiral centers, including those within amino acid side chains (i.e., Thr and Ile), were inverted (mirror‐image amino acids). The resulting CD spectra (Figure 2a) are indicative of unfolded, mirror‐image peptides. The addition of 20% trifluoroethanol, which induces the formation of α‐helices,^[^ ^36^ ^]^ yielded mirror‐image spectra with characteristic peaks at 208 and 222 nm (Figure 2a). Upon addition of *D*‐PolyU, both *L*‐Precursor‐HhH and *D*‐Precursor‐HhH formed coacervates (Figure 2b), indicating that coacervation is robust to the chiral inversion of just one partner and is thus functionally ambidextrous.

We have previously shown by electron paramagnetic resonance analysis that dimerization, RNA binding, and coacervation are linked processes.^[^ ^25^ ^]^ If true, phase separation potential may depend in part on dimerization and α‐helical folding. The α‐helical folding of Precursor‐HhH was disrupted by alternating the chirality of every other amino acid. The resulting construct, *D/L*‐Precursor‐HhH (entry 2, Table 1, and **SI** for details), exhibited almost no circular dichroism signal with or without 20% trifluoroethanol, as expected (Figure 2a). Yet, *D/L*‐Precursor‐HhH also formed coacervates upon addition of polyU (Figure 2b). Coacervation is therefore not strictly dependent on adopting the canonical HhH motif or (HhH)_2_‐Fold conformation.

### Chiral Inversion of Primordial‐(HhH)_2_

While the interactions that mediate coacervation are likely to be relatively nonspecific, the binding of Primordial‐(HhH)_2_ to the minor groove of dsDNA (Figure 1) is highly dependent on the conformation of the protein, and should therefore be sensitive to chiral inversion. After all, both biopolymers adopt higher‐order chiral conformations.

**Figure 3:**
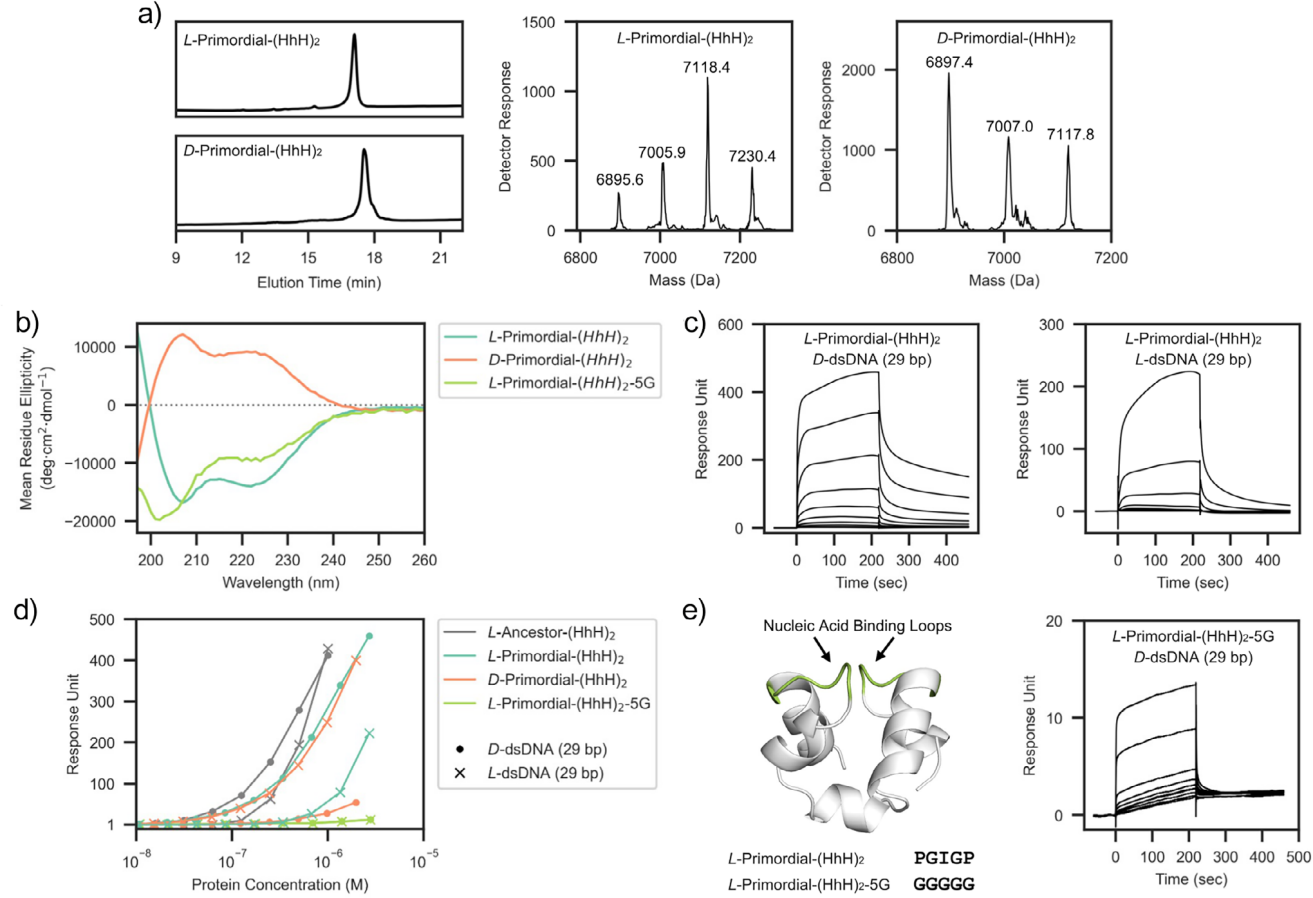
dsDNA binding of Primordial‐(HhH)_2_ in response to chiral inversion. a) *L*‐ and *D*‐Primordial‐(HhH)_2_ were synthesized by a single native chemical ligation reaction between two peptide segments, followed by a desulfurization step to convert the Cys residue at the ligation site back to Ala (as described previously,^[^ ^24^, ^25^ ^]^ see SI for details). Multiple peaks are observed due to the formation of trifluoracetic acid (TFA) adducts by the ESI (see also Figures S16–S18 for HRMS analysis). b) Circular dichroism spectra of *L*‐Primordial‐(HhH)_2_ and *D*‐Primordial‐(HhH)_2_ exhibit peaks around 208  and 222 nm, which are hallmarks of α‐helical structure. The spectra are approximately symmetric about the x‐axis, consistent with total chiral inversion and α‐helices of opposite handedness. c) SPR sensorgrams demonstrating the binding of *L*‐Primordial‐(HhH)_2_ to immobilized *L*‐dsDNA and *D*‐dsDNA (protein concentration range 5.3 nM – 2.7 µM). d) Steady‐state analysis of SPR binding data. The ability of both Primordial‐(HhH)_2_ and the more modern *L*‐Ancestor‐(HhH)_2_ to bind to either chiral form of dsDNA suggests that functional ambidexterity is a property of the fold itself, and not related to the highly simplified sequence of Primordial‐(HhH)_2_. The amount of protein bound at steady state was approximated to be 4 s prior to the end of analyte injection, 216 s in total. An analysis of the dissociation rates is presented in Figures S21 and S22. See Figure S23 for replicate SPR binding data. e) The inactivation mutant *L*‐Primordial‐(HhH)_2_‐5G, in which the canonical PGIGP binding motif (green) has been mutated to GGGGG (left). This mutant is less well‐folded than the wild‐type protein (panel b), and has greatly impaired binding affinity to dsDNA of either chirality (right, panel d; concentration range 5.4 nM–2.8 µM).

CD spectra (Figure 3b) indicate that both *D*‐ and *L*‐proteins fold into ɑ‐helical structures, with peaks around 208 and 222 nm. Just like the proteins themselves, and as expected, the CD spectra are mirror images of each other: *L*‐Primordial‐(HhH)_2_ has negative peaks (consistent with *L*‐amino acids and right‐handed ɑ‐helices) and *D*‐Primordial‐(HhH)_2_ has positive peaks (consistent with *D*‐amino acids and left‐handed ɑ‐helices). Minor differences between the spectra are likely due to the reduced purity of *D*‐amino acid reagents and inaccuracies in concentration determination.^[^ ^37^, ^38^ ^]^

### dsDNA Binding by Primordial‐(HhH)_2_ Is Functionally Ambidextrous

Binding of *D*‐Primordial‐(HhH)_2_ and *L*‐Primordial‐(Hhh)_2_ to 29 bp *D*‐dsDNA and 29 bp *L*‐dsDNA was assayed by SPR. We have previously shown that heterologously expressed *L*‐Primordial‐(HhH)_2_ binds to *D*‐dsDNA by solution‐state NMR and ELISA,^[^ ^24^ ^]^ and the SPR experiments performed here confirm that chemically synthesized *L*‐Primordial‐(HhH)_2_ also binds to *D*‐dsDNA (*L*:*D* natural chiral pair) (Figure 3c,d). Binding of the *D*‐protein to *L*‐dsDNA (*D*:*L* chiral pair) corresponds to a *mirror world*; as such, the binding affinity of these two pairs should, in principle, be identical. The resulting binding curves are consistent with this expectation,^[^ ^9^ ^]^ with the *D*‐protein:*L*‐dsDNA pair having slightly lower affinity, again likely due to some combination of inaccuracy in the protein concentrations and the lower synthetic purity of *D*‐proteins and *L*‐DNA relative to their natural counterparts.^[^ ^37^, ^38^ ^]^

Remarkably, binding of *D*‐Primordial‐(HhH)_2_ to *D*‐dsDNA (*D*:*D* chiral pair) and *L*‐Primordial‐(HhH)_2_ to *L*‐dsDNA (*L*:*L* chiral pair) was also observed (Figure 3c,d). The weaker binding of the *D*:*D* pair and the *L*:*L* pair relative to the *L*:*D* and *D*:*L* pairs is consistent with our expectation that, after nearly 4 billion years of coevolution, there should be significant optimization for the natural *L*:*D* chiral pair (and consequently for its mirror image, the *D*:*L* chiral pair). Next, we tested the binding of Ancestor‐(HhH)_2_ (entry 5, Table 1). Ancestor‐(HhH)_2_ is derived from the most probable amino acid sequence of an ancestral sequence reconstruction of the (HhH)_2_‐Fold family.^[^ ^24^ ^]^ This variant is comprised of a more complex alphabet of 15 different amino acid types, including aromatics (vs. 10 amino acid types in the Primordial‐(HhH)_2_ construct), and represents a more contemporary (HhH)_2_‐Fold domain. We observe that Ancestor‐(HhH)_2_ also binds to both *L*‐dsDNA and *D*‐dsDNA, and with an even smaller difference in affinity than the Precursor‐(HhH)_2_ constructs (Figure 3d). This observation confirms that functional ambidexterity is a characteristic of the protein fold itself, and not unique to highly sequence‐simplified variants.

Dissociation kinetics of the natural chiral pair are complex and associated with at least five phases, three of which could be satisfactorily fitted, yielding residuals of only ∼1% of the total signal (Figure S21). Although dissociation rates are largely concentration independent, at higher concentrations of *L*‐Primordial‐(HhH)_2_ the slowest of these three kinetic processes becomes slightly slower. This change may be due to cooperative binding effects, as the dsDNA becomes progressively more coated with protein molecules that can interact with each other and/or stabilize the optimal conformation of dsDNA for binding. The kinetic constants associated with the mirror‐world pair are similar (Figure S22A), as expected, though with a reduction in the fraction of signal associated with slow dissociation (Figure S22B), likely due to the reduced purity of mirror‐image molecules. Remarkably, the dissociation kinetics of the *L*‐protein:*L*‐dsDNA pair exhibit three dissociation phases that have nearly identical kinetic constants to the *L*‐protein:*D*‐dsDNA complex. The slowest dissociation phase (or phases), however, is almost completely abolished in the *L*‐protein:*L*‐dsDNA complex. Based on these data, we hypothesize the existence of multiple binding modes: at least three that are functionally ambidextrous with moderate or low affinity and at least one that is sensitive to chirality with high affinity, presumably the canonical binding mode captured in crystal structures.

To assess whether binding is mediated by the PGIGP‐binding loop and the flanking ɑ‐helices, we mutated the PGIGP loop to GGGGG, yielding the construct *L*‐Primordial‐(HhH)_2_‐5G (entry 4, Table 1). These mutations do not change the charge of the protein nor do they occlude binding by inserting bulky residues. Instead, these mutations should increase the overall flexibility of the binding loops and the protein itself, and thus disfavor binding if a near‐native conformation mediates the interaction with dsDNA. Although not as well‐folded as the parent protein (Figure 3b), *L*‐Primordial‐(HhH)_2_‐5G retains some ɑ‐helical character. As can be seen in Figure 3d,e, *L*‐Primordial‐(HhH)_2_‐5G binds to *D*‐dsDNA and *L*‐dsDNA with drastically lower affinity than any other construct tested. These results confirm that binding is not simply mediated by promiscuous charge‐charge interactions, even for the moderate and low‐affinity binding modes. The observations presented in Figure 3 are supported by data collected on a second SPR chip (Figure S23) and are in agreement with MST experiments (Figure S24), though MST experiments were concentration‐limited due to significant changes in initial fluorescence at high concentrations of DNA.

### The (HhH)_2_‐Binding Surface Is Partially Retained Upon Chiral Inversion: a Molecular Dynamics Analysis

**Figure 4:**
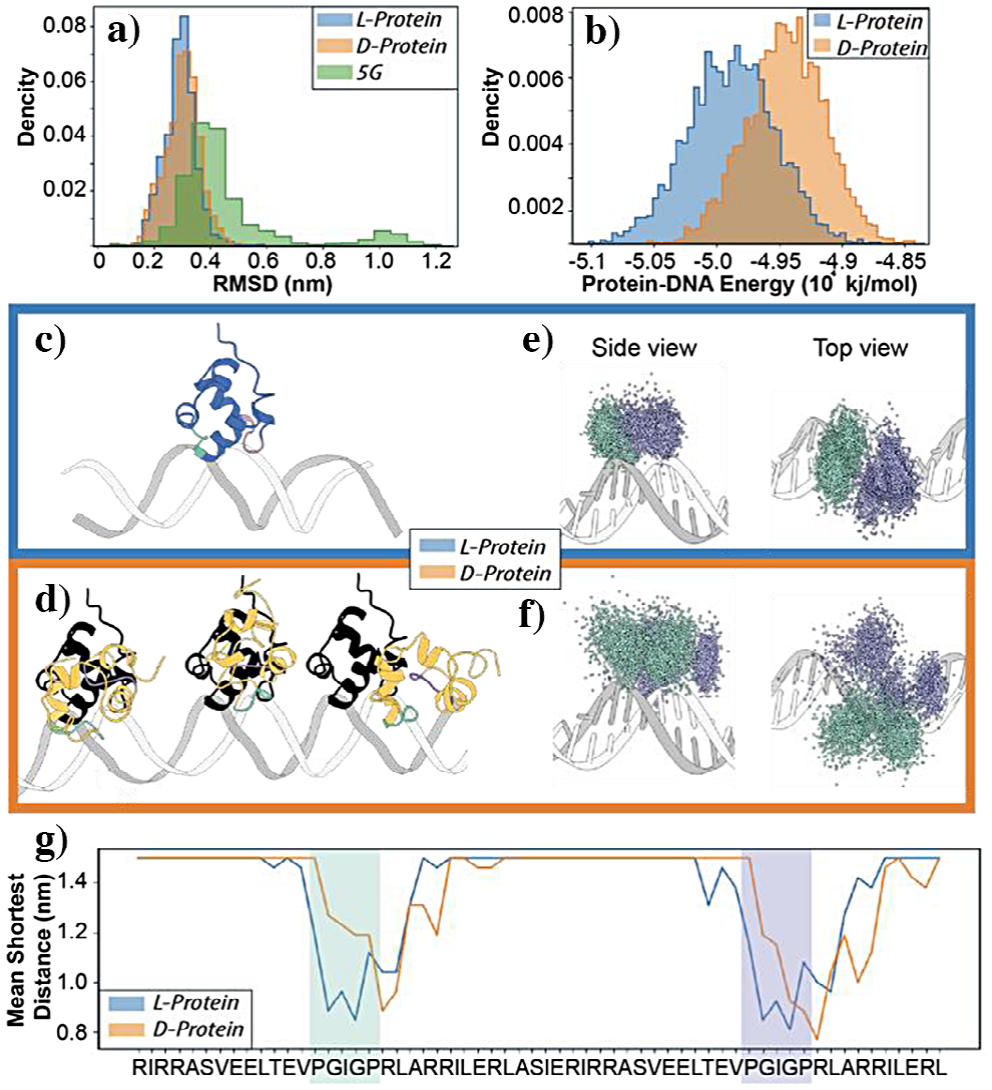
Structural and energetic analysis of (HhH)_2_‐Fold:dsDNA complexes. a) Histogram of RMSD values for *L*‐Primordial‐(HhH)₂ (*L*‐protein, *blue*), *D*‐Primordial‐(HhH)₂ (*D*‐protein, *orange*), and *L*‐Primordial‐(HhH)₂‐5G (5G, *green*). b) Distribution of total interaction energies (Lennard‐Jones + Coulomb) for *L*‐protein and *D*‐protein binding to *D*‐dsDNA. c) Representative structure from the largest cluster of the *L*‐protein system (99.9% of simulation time), colored *blue* with binding motifs in *cyan* and *purple*, aligned with the minor groove of the dsDNA. d) Representative structures of the *D*‐protein, covering 27%, 20%, and 17% of simulation time (left to right). Clustering analysis done with gmx in GROMACS. Structures are colored *orange*, with *cyan* and *purple* binding motifs. A *black* silhouette of the *L*‐protein is included for comparison. e, f) Side and top views of binding motif distributions from *L*‐protein (e) and *D*‐protein (f) systems. g) Mean shortest distance between residues and dsDNA nucleotides for *L*‐ and *D*‐proteins. Shaded regions highlight the PGIGP motifs (*cyan* and *purple*), showing that the *L*‐protein maintains consistent and close contacts, while the *D*‐protein exhibits greater variability in residue proximity, reflecting altered binding due to chiral inversion.

To assess the conformational stability and binding properties of the (HhH)_2_‐Fold variants, we simulated each complex with *D*‐dsDNA. An analysis of the root mean square deviation (RMSD) (Figure 4a) and the root mean square fluctuation (RMSF) (Figure S25) of the protein‐dsDNA complexes shows that both the *L*‐ and *D*‐Primordial‐(HhH)_2_ complexes maintained stable binding with minimal deviations from the original binding mode. *L*‐Primordial‐(HhH)_2_‐5G, however, displayed significant fluctuations, confirming its role as a negative control (Figure 3d, e). RMSF values (Figure S25) indicate that all systems exhibit low residue flexibility, suggesting that the protein structures remain stable. Notably, fluctuations were observed primarily in the N‐terminal tail (first seven residues), confirming that the HhH motifs themselves maintain structural integrity across all systems. Energetic comparisons between the systems (Figure 4b) reveal that the *L*‐protein complex achieved a lower total binding energy with *D*‐dsDNA than the *D*‐protein system, reflecting stronger binding interactions, and consistent with experiment (Figure 3d). This difference in energy distributions corresponds to greater structural deviations in *D*‐DNA binding, reflected by a small shift in RMSD values for the *D*‐protein system.

A clustering analysis of the trajectories using a 2 Å RMSD cutoff reveals a single dominant binding mode for the *L*‐protein that persists throughout nearly 100% of the simulation time (Figure 4c). This finding reinforces the strong binding configuration achieved by the native chirality. Moreover, an analysis of the *L*‐protein binding mode reveals the correct placement of the bridging sodium cation, despite the fact that the cation was not pre‐positioned at the protein/dsDNA interface (Figure 1b, Figure S26). Conversely, the *D*‐protein adopted at least three distinct binding modes (Figure 4d, left to right, corresponding to 27%, 20%, and 17% of the simulation time, respectively). This observation indicates that the *D*‐protein system has reduced conformational stability and a more transient interaction pattern as a result of the altered chirality. To further investigate the differences in binding modes, we analyzed the center of mass of the PGIGP motifs (colored *cyan* and *purple* in Figure 4e,f) across the trajectories. In the *L*‐protein system, the motifs remained densely localized within the dsDNA minor groove, forming a well‐defined interaction surface. In contrast, the *D*‐protein system showed dispersed motif localization, with the motifs shifting ∼90° along the DNA z‐axis, and creating broader, less specific contact regions outside the minor groove. These findings suggest that while the native chirality promotes precise minor‐groove binding, the inverted chirality results in more diffuse and less stable interactions. The *L*‐Primordial‐(HhH)₂‐5G variant, as expected, exhibits scattered motif positions throughout the simulation trajectory (Figure S27), confirming the absence of stable binding.

To quantify the contribution of individual residues to dsDNA binding, we calculated the mean distance between the Cα atom of each residue and the nearest DNA nucleotide (Figure 4g). This residue‐wise analysis revealed that both PGIGP motifs in the *L*‐protein (*cyan* and *purple* motifs) contribute similarly to the interaction, reflecting the conserved nature of the native HhH motif. In contrast, the *D*‐protein system showed a shift in the most contributing residues, with a slight bias toward positively charged residues at the C‐terminal side of the motif. This suggests that the inversion of chirality impacts the structural alignment of the motifs, leading to altered residue contributions. Nevertheless, the PGIGP motif clearly contributes to binding in both cases.

In summary, the *L*‐protein relies more heavily on the precise configuration of its HhH motifs for stable binding, whereas the *D*‐protein compensates for structural misalignment by increasing reliance on electrostatic interactions from positively charged residues. These observations are consistent with the energy calculations and cluster analysis, which show that the *D*‐protein adopts multiple binding modes with weaker stability. The MD results also indicate that both *L*‐ and *D*‐Primordial‐(HhH)_2_ interact with *D*‐dsDNA with higher affinity than Primordial‐(HhH)₂‐5G. These results are broadly consistent with an affinity ranking from experimental data (Figure 3) and the observation of multiple dissociation phases for the *D*‐Primordial‐(HhH)_2_:*D*‐dsDNA pair (Figures S21 and S22). The correct positioning of the bridging sodium ion^[^ ^33^ ^]^ for the natural binding pair (Figure S26) further supports the accuracy of the MD simulations. Finally, the MD data are consistent with at least partial involvement of the conserved binding surfaces between the natural and unnatural chiral pairs (Figure 4g) as also suggested by the very weak binding of Primordial‐(HhH)₂‐5G (Figure 3e).

### Ambidexterity, Constraint, and the Evolution of Nucleic Acid Binding

Total chiral inversion was tolerated along the entire putative evolutionary trajectory of the HhH protein family – from coacervation of a single HhH peptide with RNA to binding of the (HhH)_2_‐Fold to dsDNA. These results suggest that functional ambidexterity is an enduring feature of this fold. Previously, Kay and coworkers reported that GroEL/ES was functionally ambidextrous with respect to the protein substrate,^[^ ^20^ ^]^ as it was able to refold DapA of either chirality into an active conformation. We hypothesize that the GroEL/ES result can be rationalized as a consequence of the non‐specific interactions made by chaperones and their need to accommodate diverse protein substrates. Likewise, the robustness to coacervation upon chiral inversion of the HhH motif is consistent with this motif forming relatively non‐specific side‐chain interactions to RNA^[^ ^25^ ^]^– so much so that dimerizing and folding into the canonical (HhH)_2_‐Fold is not required (though oligomerization into some other form may be possible).

For dsDNA binding by the (HhH)_2_‐Fold, a somewhat different story emerges, as the binding interaction is sensitive to the conformation (and conformational stability) of the protein, as demonstrated by the dramatically reduced binding of *L*‐Primordial‐(HhH)_2_‐5G. Moreover, binding of both the natural and cross‐chiral pairs is mediated by a partially overlapping surface. Given that essentially one chiral form of dsDNA and protein has existed on Earth for billions of years, there was little pressure to evolve specificity against mirror‐image biopolymers. Ambidextrous binding modes, thus, may stem from unrelated functional constraints, such as a need to interact with ssDNA or dsDNA of various conformations, to promote sliding along the dsDNA, or as the transient mode of interaction that promotes partitioning of molecules near the charged surface of dsDNA. It is therefore tantalizing to envision that lower‐affinity, ambidextrous binding modes represent a more primitive or ancestral mode of binding.

## Conclusions

The HhH motif and (HhH)_2_‐Fold are ancient and ubiquitous nucleic‐acid binders. We now report that both the HhH motif and the (HhH)_2_‐Fold exhibit signatures of functional ambidexterity, in which chiral inversion of one binding partner *does not abolish binding*. We conclude that chiral inversion experiments can provide a unique window into the interplay between the high‐ and low‐specificity modes of interaction that underlie nucleic acid binding in this and other ancient protein families.

## Supporting Information

Supplementary materials and methods, including synthesis and characterization of peptides and proteins, are available online.

The authors have cited additional references within the Supporting Information.^[^ ^39^, ^40^, ^41^, ^42^, ^43^, ^44^, ^45^, ^46^, ^47^ ^]^

## Conflict of Interests

The authors declare no conflict of interest.

## Supporting information



Supporting Information

## Data Availability

The data that support the findings of this study are available in the supplementary material of this article.
