# Supplementary material for: Functional Ambidexterity of an Ancient Nucleic Acid‐Binding Domain
Source: Angew Chem Int Ed Engl. 2025 May 8;64(25):e202505188. doi: 10.1002/anie.202505188 (PMC12171331; doi:10.1002/anie.202505188)
Supplement: Supplementary file 1 — Supporting Information [file ANIE-64-e202505188-s001.pdf]

Supplemental Information

Functional Ambidexterity of an Ancient Nucleic Acid-Binding Domain

Orit Weil-Ktorza‡,[1], Segev Naveh-Tassa‡,[2], Yael Fridmann-Sirkis‡,[3], Dragana Despotović‡,[4,5], Kesava Phaneendra Cherukuri[4], Tatsuya Corlett[6], Yaakov Levy[2],*, Norman Metanis[1],*, Liam M. Longo[6,7],*

[1] Dr. Orit Weil-Ktorza, Prof. Norman Metanis
Institute of Chemistry, The Center for Nanoscience and Nanotechnology, Casali Center of Applied Chemistry
The Hebrew University of Jerusalem
Jerusalem 9190401, Israel
E-mail: metanis@mail.huji.ac.il

[2] Mr. Segev Naveh-Tassa, Prof. Yaakov Levy
Department of Chemical and Structural Biology
Weizmann Institute of Science
Rehovot 7610001, Israel
E-mail: koby.levy@weizmann.ac.il

[3] Dr. Yael Fridmann-Sirkis
Department of Life Sciences Core Facilities
Weizmann Institute of Science
Rehovot 7610001, Israel

[4] Dr. Dragana Despotović, Dr. Kesava Phaneendra Cherukuri
Department of Biomolecular Sciences
Weizmann Institute of Science
Rehovot 7610001, Israel

[5] Dr. Dragana Despotović
Institute of Molecular Genetics and Genetic Engineering
University of Belgrade
Belgrade 11042, Serbia

[6] Prof. Liam M. Longo, Mr. Tatsuya Corlett
Earth-Life Science Institute
Institute of Science Tokyo
Tokyo 152-8550, Japan
E-mail: llongo@elsi.jp

[7] Prof. Liam M. Longo
Blue Marble Space Institute of Science
Seattle, Washington 98104, USA

[‡] These authors contributed equally to this work.

Supplemental Materials and Methods

Reagent sourcing

Buffers were prepared using MilliQ water (Millipore, Merck). Ultrapure guanidinium chloride (Gn·HCl, Chem-Impex Inc) was used in all ligation reactions. $Na_2HPO_4·12H_2O$, ethanedithiol (EDT), triisopropylsilane (TIPS), 4-mercaptophenylacetic acid (MPAA) were purchased from Sigma-Aldrich (Rehovot, Israel). Cy5-NHS ester was purchased from BroadPharm® (USA). Acetylacetone and tris(2-carboxyethyl)phosphine (TCEP) were purchased from Tokyo Chemical Industry. *p*-nitrophenyl chlorophormate was purchased from Acros Organics. All Fmoc-amino acids (*D*- and *L*-) were obtained from CS Bio Co. (Menlo Park, CA), Matrix Innovation (Quebec City, Canada) or Chem-Impex Inc., with the following side chain protecting groups: Arg(Pbf), Glu(OtBu), Gly(OtBu), Ser(tBu), Thr(tBu), (Pbf = 2,2,4,6,7-pentamethyl-2,3-dihydrobenzofuran-5-sulfonyl). TentaGel® R RAM resin (loading 0.19 mmol/g) was purchased from Rapp Polymer GmbH (Germany) and 2-chlorotrityl-resin (loading of 0.3-0.8 eq/g) was purchased from Chem-Impex Inc. 1-[Bis(dimethylamino)methylen]-5- chlorobenzotriazolium 3-oxide hexafluorophosphate and *N,N,N',N'*-Tetramethyl-O-(6-chloro-1H-benzotriazol-1-yl)uronium hexafluorophosphate (HCTU) were purchased from Luxembourg Biotechnologies Ltd. (Rehovot, Israel). All solvents: *N,N*-dimethylformamide (DMF), dichloromethane (DCM), acetonitrile (ACN), *N,N*-diisopropylethyl amine (DIEA), Trifluoroacetic acid (TFA), piperidine (Pip), dimethylsulfoxide (DMSO) and Boc-anhydride were purchased from Bio-Lab (Jerusalem, Israel) and were peptide synthesis, HPLC or ULC-grade.

High Performance Liquid Chromatography (HPLC)

Analytical reversed-phase HPLC (RP-HPLC) was performed on a Waters Alliance HPLC with 220 and 280 nm UV detection using an XBridge BEH300 C4 column (3.5 µm, 130 Å, 4.6 × 150 mm). Semi-preparative RP-HPLC was performed on a XBridge BEH C4 column (5 µm, 300 Å, 10 × 150 mm) and XSelect CSH C18 column (5 µm, 130 Å, 10 × 150 mm). Preparative RP-HPLC was performed on a XSelect C4 column (5 µm, 130 Å, 19× 250 mm) or XSelect C18 column (5 µm, 30 × 250 mm). The flow rates were 1 mL/min (analytical), 3.35 mL/min (semi-preparative),

or 10-20 mL/min (preparative). Linear gradients of ACN (with 0.1% TFA, eluent B) in water (with 0.1% TFA, eluent A) were used for all systems to elute bound peptides.

Electrospray Ionization Mass Spectrometry (ESI-MS)

ESI-MS was performed on LCQ Fleet Ion Trap mass spectrometer (Thermo Scientific). Peptide masses were calculated from the experimental mass to charge (m/z) ratios from the observed multiply charged species of a peptide. Deconvolution of the experimental MS data was performed with MagTran v1.03.

High-Resolution Mass Spectrometry (HR-MS)

HR-MS spectra were recorded on a Q Exactive Plus Orbitrap mass spectrometer (Thermo Scientific) with an ESI source and 140,000 FWHM, in a method with the automatic gain control (AGC) target set to 1E6 and a scan range of 400-2800 m/z. Deconvolution of the raw MS data was performed with MagTran v1.03.

Circular Dichroism

Circular dichroism (CD) spectra were collected on a Chirascan CD spectrometer (Applied Photophysics) with a 1-mm pathlength quartz cuvette. Samples containing 10 µM *L*-Precursor-HhH, *D*-Precursor-HhH or *D/L*-Precursor-HhH were measured in 5 mM Tris·HCl, 50 mM NaCl, pH 7.5 with either 0% or 20% (v/v) trifluoroethanol (TFE). Samples containing 5 µM *L*-Primordial-$(HhH)_2$, *D*-Primordial-$(HhH)_2$ or *L*-Primordial-$(HhH)_2$-5G were measured in 5 mM Tris·HCl, 500 mM NaCl, 10 mM $MgCl_2$, 5 mM $CaCl_2$, pH 7.5. All spectra were collected from 195 to 260 nm with a data pitch of 1 nm at room temperature, adaptive sampling (variable signal averaging at each wavelength), and a slit width of 1 nm. The photomultiplier tube voltage during measurement was kept below 700 V, and data points exceeding this value were discarded. All reported spectra have had the spectrum of the buffer subtracted.

Phase Separation

Peptides and polyuridylic acid (polyU; Sigma-Aldrich, P9528) were dissolved in Milli-Q water (Millipore Sigma). Peptide concentrations were measured using the Pierce BCA Protein Assay Kit (Thermo Fisher Scientific). Stock solutions of 10 mg/mL polyU and 500 mM MES pH 5.6 were prepared. Phase separation was induced by mixing the peptide and polyU solutions. The final composition of the phase separation reaction mixture was 50 mM MES pH 5.6, 1.0 mg/mL polyU, and 300 μM peptide. Typically, 3 μL of the phase separation reaction mixture was loaded onto slides (24 × 40 mm, 0.13-0.16 mm thick) and observed using an Eclipse TI-E Nikon inverted microscope (Nikon Instruments Inc., Melville, NY) with an oil-immersion 100× objective (Plan Apo, 100×/1.40 oil). Images were acquired with a cooled electron-multiplying charge-coupled device camera (IXON ULTRA 888, Andor). Pictures were analyzed using the Fiji platform.[1]

Surface Plasmon Resonance

Binding of 29-bp *D*-dsDNA and *L*-dsDNA (the mirror-image DNA derived from *L*-ribose; see **Table S1** for DNA sequences) was monitored by surface plasmon resonance (SPR) on a Biacore S200 system (Cytiva, Sweden). Since the $(HhH)_2$-Fold variants are positively charged at neutral pH, a C1 chip (S-Series Cytiva, Sweden), which carries less negative charge than the standard CM5 chip, was used. Streptavidin was conjugated to the chip surface using EDC/NHS chemistry in the presence of acidified sodium acetate buffer (150 mM, pH 3.8), as outlined in the C1 sensor chip manual. Approximately 2,000 RU (Chip 1, **Main Text**; buffer pH 3.8) or 700 RU (Chip 2, **SI**; buffer pH 4.6) of streptavidin was covalently conjugated to the chip surface and then blocked by injecting 1 M ethanolamine pH 8.0 for 5 min. Subsequently, 405 RU of *D*-dsDNA and 423 RU of *L*-dsDNA (Chip 1) or 95 RU of *D*-dsDNA and 97 RU of *L*-dsDNA (Chip 2) in which one strand of the duplex was 5'-biotinulated was stably associated to the surface of one channel. Before data collection, a normalization cycle followed by three priming cycles were run to stabilize the instrument. Binding assays were performed in SPR binding buffer (50 mM Tris, 150 mM NaCl, 0.05% Tween-20, pH 7.5) with a flow rate of 20 μL/min at 25 °C. Regeneration of the chip surface was achieved by a 60 s injection of 2 M NaCl in water. Reported sensorgrams were double subtracted: First, by the background binding of the analyte to a streptavidin-conjugated control channel and then by the average of 2 buffer injection runs.

Microscale Thermophoresis (MST)

Protein-DNA interactions were analyzed by microscale thermophoresis. Experiments were performed with 25 nM of synthetic, Cy5-labelled $(HhH)_2$-Fold proteins, which were prepared by coupling Cy5-NHS ester to the N-terminus of synthetic proteins (note that there are no Lys residues present in these sequences). Experiments were carried out in a microMonolith NT.115 Blue/Red (NanoTemper Technologies) at 25 °C. Labelled peptides were mixed with serially diluted DNA samples in 50 mM Tris, 150 mM NaCl, 0.05% Tween-20, pH 7.5 in premium capillaries (NanoTemper Technologies) at 40% MST power. Dissociation constants (K_D) could not be calculated for a 1:1 binding model as binding is non-specific for the minor groove of dsDNA and, as a result, one strand of dsDNA has many degenerates, overlapping binding sites. See **Table S1** for DNA sequences.

Molecular Dynamics (MD) Simulations

To investigate the differences in binding stability between the native *L*-chirality and the inverted *D*-chirality, we conducted molecular dynamics (MD) simulations on three systems: *L*-Primordial-$(HhH)_2$, *D*-Primordial-$(HhH)_2$, and *L*-Primordial-$(HhH)_2$-5G (negative control). The simulations were performed using GROMACS 2022[2] with the CHARMM36[3] force field. Each system was placed in a dodecahedral periodic simulation box with dimensions of 9 × 7 × 5 nm, ensuring a minimum distance of 1.0 nm between the solute and the box edges. The systems were solvated in a TIP3P water box with 0.125M NaCl to maintain charge neutrality.

After energy minimization using the steepest descent algorithm, the systems were equilibrated with NVT and NPT ensembles[4]. Periodic boundary conditions were applied in all three dimensions. The Particle Mesh Ewald (PME) method was used to compute long-range electrostatic interactions with a Fourier spacing of 0.12 nm and a tolerance of 10^{-5}. Short-range electrostatic and van der Waals interactions were truncated at a 1.0 nm cutoff using a Verlet neighbor list, updated every 20 steps.

Production simulations were performed at 300K for 1 µs per run and repeated three times to ensure robust sampling. Each of the three independent MD trajectories was initiated from the same

minimized and equilibrated structure but with different initial velocities, generated from a Maxwell-Boltzmann distribution at 300K, to ensure independent sampling of conformational space. A time step of 2 fs was used, and non-bonded interactions were calculated with a 1 nm cutoff.

The *L*-Primordial-$(HhH)_2$ structure was generated using AlphaFold2, yielding high-confidence predictions with most residues scoring above 80 on the IDDT plot (**Figure S28**). This structure was aligned to a 60-residue sequence from the 1C7Y PDB template using PyMOL. *D*-Primordial-$(HhH)_2$ was generated through DStabilize[5], and *L*-Primordial-$(HhH)_2$-5G was produced via mutagenesis in PyMOL. The DNA sequence was extended to 21 base pairs using chains C and G from the 1C7Y template. During production runs, positional restraints were applied to one atom at the end of the DNA strand to mimic a longer linear DNA molecule.

Chemical Protein Synthesis

Proteins and peptides characterized in this study were prepared by chemical synthesis using solid-phase peptide synthesis (SPPS)[6] and native chemical ligation (NCL)[7] followed by desulfurization[8] of the Cys residue at the ligation site, as described previously[9] (see below a detailed protocol and **Figures S1-S19** and **Schemes S1** and **S2**). Construct names and sequences are given in **Table 1 (**in the **Main Text**).

Peptide Synthesis

General procedure for Fmoc-Solid Phase Peptide Synthesis (SPPS): Peptides were prepared by automatic peptide synthesizer (CS136XT, CS Bio Inc. CA) typically on 0.25 mmol scales. Fmoc-protected amino acids (2 mmol in 5 mL DMF) were activated with HCTU (2 mmol in 5 mL DMF) and DIEA (4 mmol in 5 mL DMF) for 5 min and allowed to couple for 25 min, with constant shaking. Fmoc-deprotection was carried out with 20% piperidine in DMF (2 × 5 min).

Cleavage, deprotection and purification: The peptide-resins were washed with DMF and DCM and then dried under vacuum. The dried peptide-resins were deprotected and simultaneously cleaved using a TFA/water/thioanisole/triisopropylsilane/ethanedithiol (92.5:1.5:1.5:1.5:1.5)

cocktail for 4 h. The cleavage mixtures were filtered and TFA was evaporated with N_2-bubbling to a minimum volume, to which an eightfold volume of cold ether was added dropwise. The precipitated crude peptides were centrifuged (5,000 rpm, 10 min), ether was removed, and the crude peptide was dissolved in ACN/water (1:1) containing 0.1% TFA and was further diluted to ca. 25% ACN with water and lyophilized.

Synthesis of *L*-Primordial-(HhH)$_2$

Sequence:

RIRRASVEELTEVPGIGPRLARRILER**LA**SIERIRRASVEELTEVPGIGPRLARRILERL

L-Primordial-(HhH)$_2$ was prepared from two peptide segments using the native chemical ligation (NCL) and desulfurization approach (**Scheme S1**). The two peptide segments were the thioester surrogate *L*-Primordial(1-28)-Nbz (Nbz = *N*-acylurea)[10], and *L*-Primordial(29-60)(A29C), in which Ala29 was temporarily substituted with Cys to allow for the NCL reaction, and was later desulfurized to natural Ala29 post-ligation. The ligation site within the sequence (above) is shown in bold and underlined.

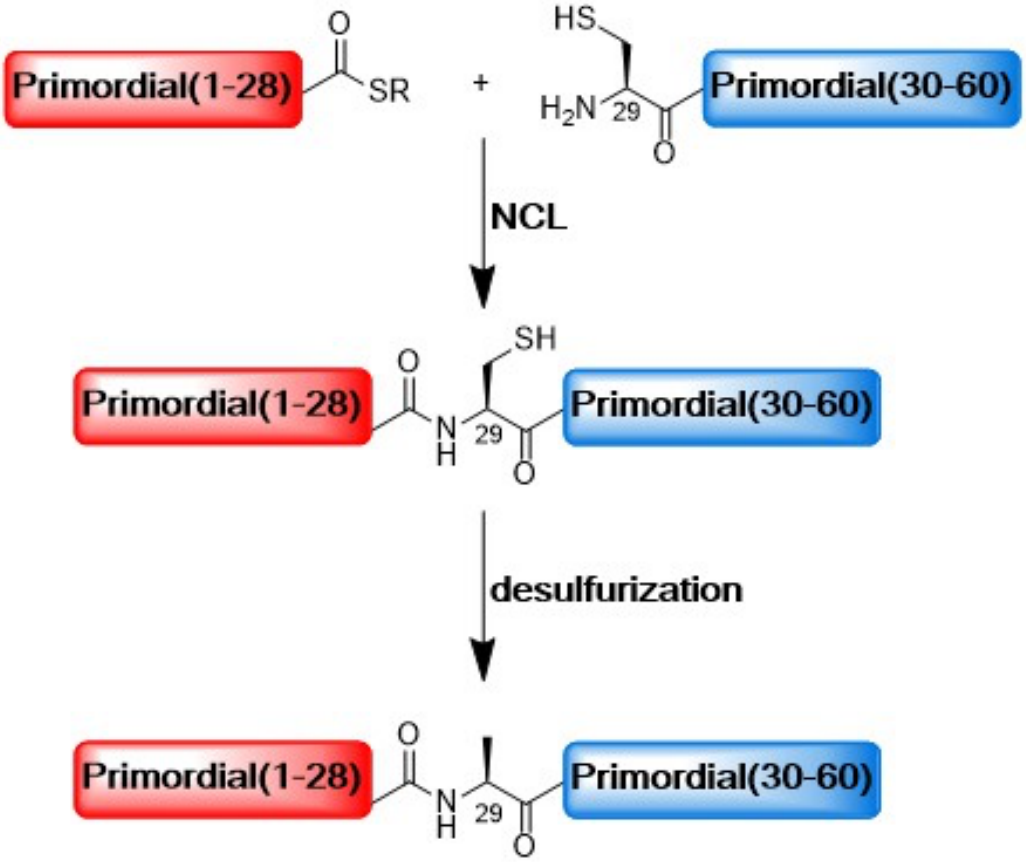

Scheme S1. **Synthesis of *L*-Primordial-(HhH)$_2$**. The protein was synthesized from two half-peptides and then joined using NCL and desulfurization. The N-terminal half-peptide bears a C-terminal thioester surrogate (shown here as thioester for simplicity), and the C-terminal peptide bears an N-terminal Cys residue. After peptide ligation, the Cys residue is desulfurized to yield alanine.

Synthesis of L-Primordial(1-28)-Nbz peptide:

L-Primordial(1-28)-Nbz was synthesized first on Fmoc-Dbz-resin (0.25 mmol scale)[10] with an automated peptide synthesizer. Mono-Fmoc-3,4-diaminobenzoic acid (Fmoc-Dbz-OH, 3 equiv)[10] was activated with HCTU (3 equiv)/DIEA (6 equiv) in DMF and was doubly coupled to the free amine of TentaGel® R RAM resin (0.19 mmol/g, 0.25 mmol scale) for 1 h. The first amino acid, Leu28, was also doubly coupled.

N-terminal Boc-protection: After synthesis completion, the Fmoc protecting group of the N-terminal Arg was removed and the peptide-resin was treated with 1.5 equiv of Boc-anhydride solution dissolved in 10 mL DCM and 2.0 equiv DIEA. The reaction was left overnight to give N-terminal Boc-protected peptide-resin required before the step of Dbz to Nbz conversion.

On-resin Nbz formation: The resin was washed with DCM and a solution of *p*-nitrophenyl chloroformate (5 equiv, 1.25 mmol) in DCM (5 mL) was added, shaken for 1 h at 25 °C and washed with DCM (3 × 5 mL) and DMF (3 × 5 mL). This step was repeated one more time. Following this, the resin was washed with DMF and a 5 mL solution of 0.5 M DIEA in DMF was added and shaken for an additional 30 min to complete the cyclization/Nbz formation (repeated twice), and washed with DMF (3 × 5 mL) and DCM (3 × 5 mL) and dried under vacuum.

Deprotection and cleavage: The peptide-resin was deprotected and cleaved as described previously to give 909 mg of crude *L*-Primordial(1-28)-Nbz.

Purification of L-Primordial(1-28)-Nbz: 150 mg of crude peptide were taken and dissolved in 25% ACN with water and purified by preparative RP-HPLC (XSelect C18 column, 5 µm, 130 Å, 30 × 250 mm), using a gradient of 30-60% B over 42 min to give pure *L*-Primordial(1-28)-Nbz (21 mg, 15% yield). The HPLC analysis (**Figure S1**) was carried out on a C4 analytical column (XBridge BEH300, 3.5 µm, 130 Å, 4.6 × 150 mm) using a gradient of 5% B over 2 min then 5-70% B over 20 min).

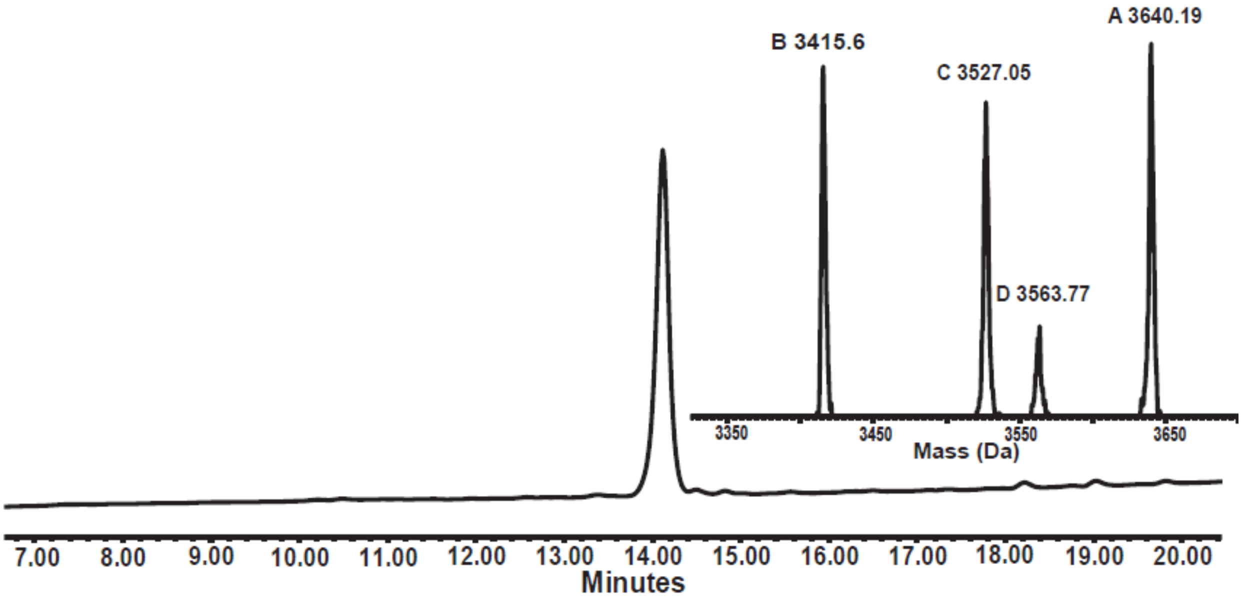

Figure S1. HPLC chromatograms and ESI-MS for purified *L*-Primordial(1-28)-Nbz, with the inset showing the corresponding mass (calc. 3416.0 Da; obs. 3415.6 Da, also observed [M+1 TFA] 3527.05 Da, [M+2 TFA] 3640.19 Da; the 3563.77 Da species is an impurity).

Synthesis of the second segment, L-Primordial(29-60)(A29C):

The peptide was synthesized on 2-chlorotrityl-resin (loading 0.3 mmol/g, 0.25 mmol scale) on an automated peptide synthesizer. Ala29 was substituted with Cys to permit Cys-NCL with the first segment *L*-Primordial(1-28)-Nbz.

Deprotection and cleavage: Peptide was deprotected and cleaved as described previously to give 1356 mg of crude *L*-Primordial(29-60)(A29C).

Purification: The peptide was purified by RP-HPLC (200 mg of crude) (XSelect C18 column, 5 µm, 130 Å, 30 × 250 mm) using a gradient of 30-60% B over 42 min to give pure Primordial(29-60)(A29C) (68 mg, 34% yield). The HPLC analysis (**Figure S2**) was carried out on a C4 analytical column.

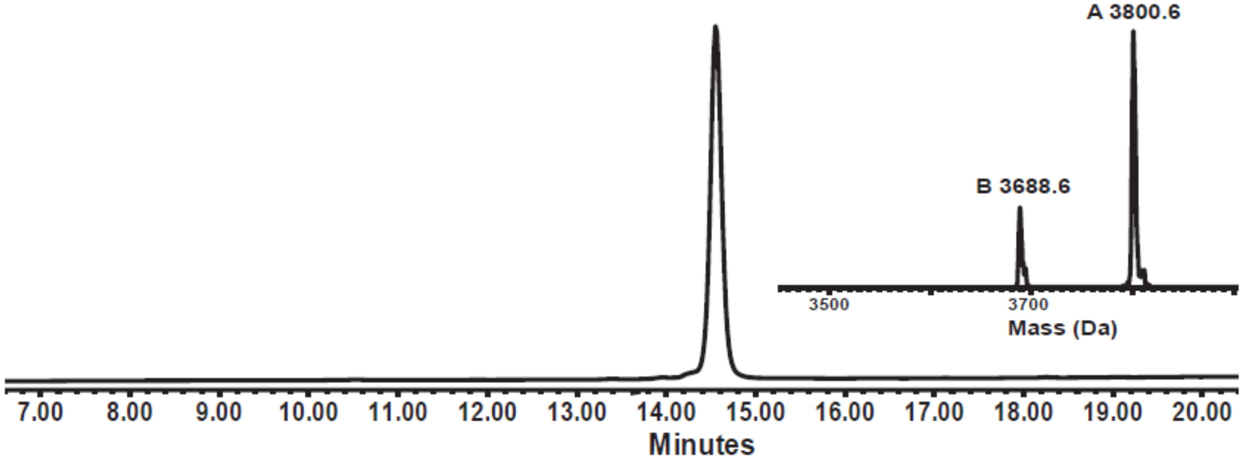

Figure S2. HPLC chromatograms and ESI-MS for purified *L*-Primordial(29-60)(A29C), with the inset showing the corresponding mass (calc. 3689.3 Da; obs. 3688.6 Da, [M+TFA] 3800.6 Da).

NCL between L-Primordial(1-28)-Nbz and L-Primordial(29-60)(A29C): For ligation, *L*-Primordial(1-28)-Nbz peptide (~10.5 mg, 3.1 µmol, final conc. ~1 mM) was dissolved in 3 mL of argon-degassed phosphate buffer (200 mM NaH_2PO_4, 6 M Gn·HCl, 0.2 M MPAA, 0.05 M TCEP , pH 6.5) and this mixture was added to *L*-Primordial(29-60)(A29C) peptide (~16 mg, 4.34 µmol, final conc. ~1.3 mM). The progress of the reaction was followed by analytical HPLC (XBridge C4 column, 3.5 µm, 4.6 × 150 mm) with a gradient of 10-60% B over 20 min. The ligation was quenched after 4 h. The ligation product was purified by semi-preparative HPLC (XBridge BEH300 C4 column, 5 µm, 19 × 150 mm, method 25-50% B over 45 min) to yield the corresponding *L*-Primordial(1-60)(A29C)-$(HhH)_2$ product (10 mg, 38% yield; **Figure S3**).

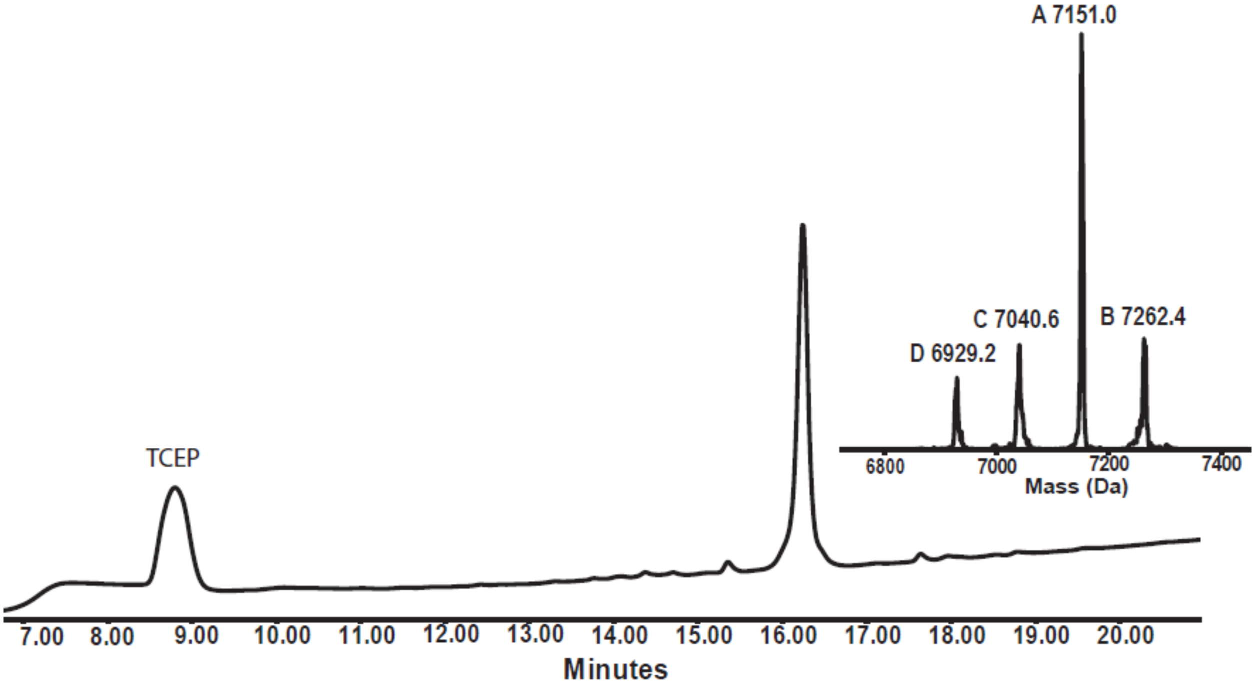

Figure S3. HPLC chromatograms and ESI-MS for purified *L*-Primordial-(1-60)(A29C)-$(HhH)_2$, with the inset showing the corresponding mass (calc. 6928.20 Da; obs 6929.2 Da, [M+TFA] 7040.6 Da, [M+2TFA] 7151.0 Da, [M+3TFA] 7262.4 Da).

Desulfurization of L-Primordial(1-60)(A29C)-$(HhH)_2$ to give ***L-Primordial-$(HhH)_2$****:* *L*-Primordial(1-60)(A29C)-$(HhH)_2$ (17 mg, 2.46 µmol) was dissolved in 4 mL of argon degassed phosphate buffer (200 mM NaH_2PO_4, 6 M Gn·HCl, 400 equiv TCEP, 200 equiv VA-044, pH 5.5), and left for 48 hours to afford the desired product. The progress of the reaction (**Figure S4**) was followed by analytical HPLC (XBridge C4 column, 3.5 µm, 4.6 × 150 mm) with a gradient 10-60% B over 20 min. *L*-Primordial-$(HhH)_2$ was purified by semi-preparative HPLC (XBridge BEH300 C4 column, 5 µm, 19 × 150 mm, method 25-50% B over 45 min) (8 mg, 47% yield).

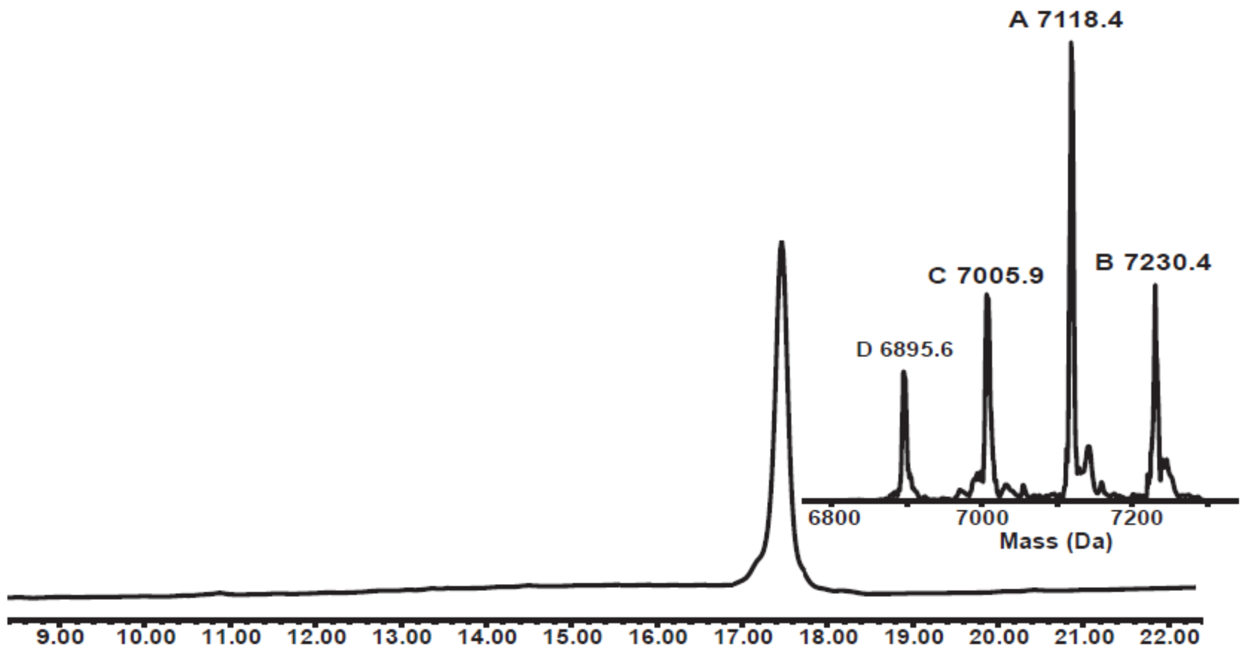

Figure S4. Desulfurization of *L*-Primordial-(1-60)(A29C)-(HhH)$_2$. Analytical HPLC of the desulfurization reaction after 48 h with the ESI-MS of desired product *L*-Primordial-(HhH)$_2$ (calc. 6896.1 Da; obs. 6895.6 Da, [M+TFA] 7005.9 Da, [M+2 TFA] 7118.4 Da, [M+3 TFA] 7230.4 Da).

Synthesis of *D*-Primordial-(HhH)$_2$

***Sequence (same as the L-Primordial-(HhH)$_2$)*:**

rirrasveeltevpgigprlarrilerl**<u>la</u>**sierirrasveeltevpgigprlarrilerl

The *D*-form of the Primordial-(HhH)$_2$ protein was prepared using the same approach as for the *L*-protein, from two peptide segments using NCL and desulfurization (**Scheme S1**). The two peptide segments were *D*-Primordial(1-28)-NHNH$_2$, and *D*-Primordial(29-60)(A29C), in which Ala29 was temporary substituted with Cys (in the form of *D*-Cys) to allow for the NCL reaction, and was then desulfurized to Ala29 after ligation. The ligation site within the sequence (above) is shown in bold and underlined.

Synthesis of D-Primordial(1-28)-COSR peptide:

For the synthesis of *D*-Primordial(1-28)-COSR, we first prepared the C-terminal hydrazide thioester surrogate, which was carried out on 2-chlorotrityl chloride-resin. First, the resin (0.25 mmol scale; loading 0.5 mmol/g) was swelled in DMF for 1 h and treated twice with freshly

prepared 5% hydrazine in DMF for 1 h and decanted[11]. The resin was washed well with DMF and then treated with 10% MeOH in DMF for 30 min. The hydrazine functionalized chlorotrityl-resin was used for standard Fmoc-SPPS, with the coupling of the *D*-amino acids performed using automated synthesizer, while the *D*-Ile residues were manually coupled.

Deprotection and cleavage: Peptide was deprotected and cleaved off the resin as described previously to give 400 mg of crude *D*-Primordial(1-28)-$NHNH_2$.

Purification: The peptide was purified by RP-HPLC (100 mg of crude peptide) on an XSelect C18 column (5 µm, 130 Å, 30 × 250 mm) using a gradient of 30-60% B over 42 min to give pure *D*-Primordial(1-28)-$NHNH_2$ (15 mg, 15% yield). The HPLC analysis (**Figure S5**) was carried out on a C4 analytical column.

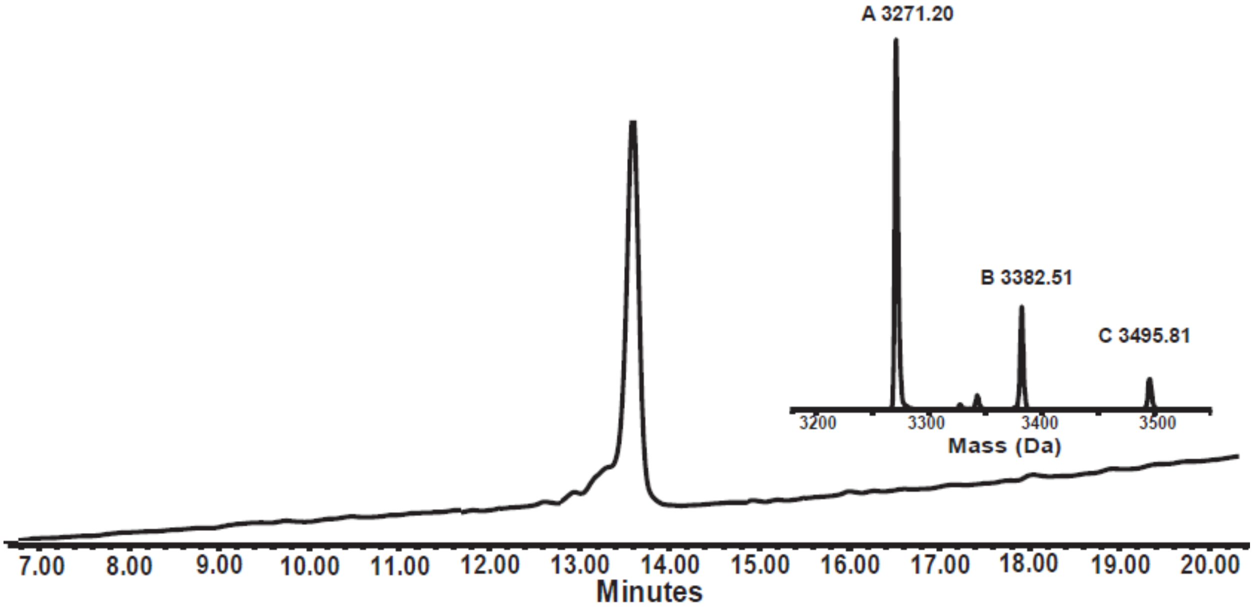

Figure S5. HPLC chromatograms and ESI-MS for purified *D*-Primordial(1-28)-$NHNH_2$, with the inset showing the corresponding mass (calc. 3270.89 Da; obs. 3271.20 Da, [M+TFA] 3382.51 Da, [M+2 TFA] 3495.81 Da).

Procedure for thioesterification

The conversion to thioester was done by dissolving 15 mg of peptide in phosphate buffer (200 mM, 6 M Gn·HCl, pH ~2.5; final conc. ~1 mM) and treated with 50 equivalents of acetylacetone

(acac)[12] and 200 equiv of MPAA for 2 h at room temperature. This reaction mixture was kept for the next ligation step.

Synthesis of the second segment, D-Primordial(29-60)(A29C):

This peptide was synthesized on an automated synthesizer with manual coupling of *D*-Ile residues and cleaved as described previously for *L*-Primordial(29-60)(A29C) to obtain 405 mg of crude *D*-Primordial(29-60)(A29C).

Purification: The peptide was purified by RP-HPLC (100 mg of crude) (XSelect C18 column, 5 µm, 130 Å, 30 × 250 mm) using a gradient of 30-60% B over 42 min to give pure *D*-Primordial(29-60)(A29C) (26 mg, 26% yield). The HPLC analysis (**Figure S6**) was carried out on a C4 analytical column.

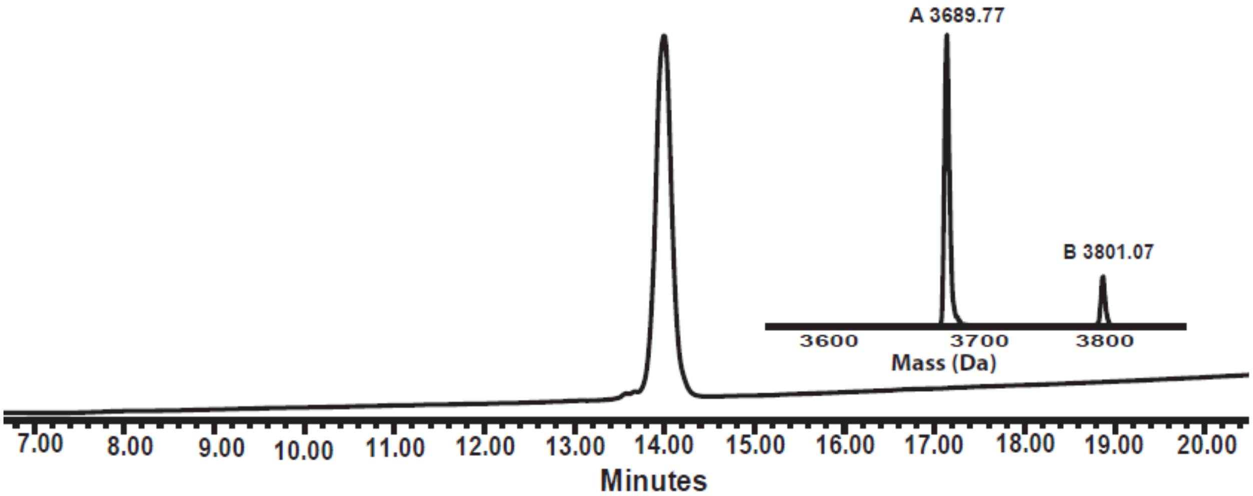

Figure S6. HPLC chromatograms and ESI-MS for purified *D*-Primordial(29-60)(A29C), with the inset showing the corresponding mass (calc. 3689.35 Da; obs. 3689.77 Da, [M+TFA] 3801.07 Da).

NCL between D-Primordial(1-28)-COSR and D-Primordial(29-60)(A29C): For the ligation reaction, 200 equivalents of TCEP were added in the thioesterification reaction mixture and the pH was adjusted to 6.5. Then, *D*-Primordial(29-60)(A29C) peptide (~22.5 mg, 6.1 µmol, final conc. ~1.3 mM) was added. The progress of the reaction was followed by analytical HPLC (XBridge C4 column, 3.5 µm, 4.6 × 150 mm) with a gradient of 10-60% B over 20 min. The ligation was left overnight. Ligation product was purified by semi-preparative HPLC (XBridge

BEH300 C4 column, 5 µm, 19 × 150 mm, method 25-50% B over 45 min) to afford the corresponding *D*-Primordial(1-60)(A29C)-(HhH)$_2$ (7 mg, 19% yield; **Figure S7**).

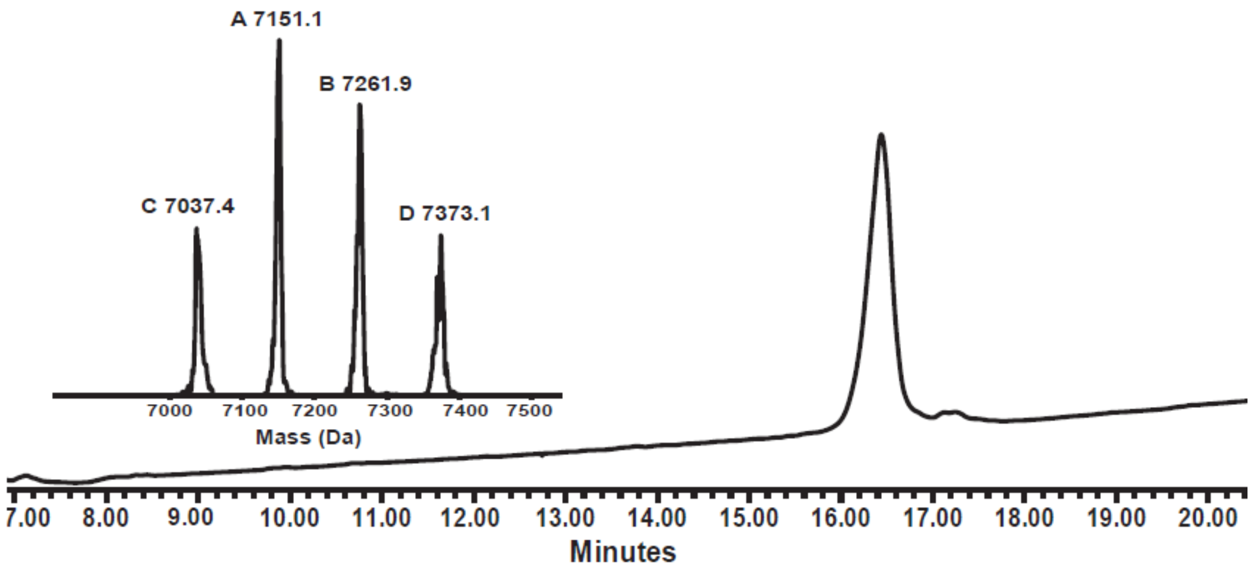

Figure S7. HPLC chromatograms and ESI-MS for purified *D*-Primordial(1-60)(A29C)-(HhH)$_2$, with the inset showing the corresponding mass (calc. 6928.2 Da, [M+TFA] 7042.2 Da; obs. [M+TFA] 7037.4 Da, [M+2 TFA] 7151.1 Da, [M+3 TFA] 7261.9 Da, [M+4 TFA] 7373.1 Da).

Desulfurization of D-Primordial(1-60)(A29C)-(HhH)$_2$ to give ***D-Primordial-(HhH)$_2$**:* The desulfurization reaction was performed as described previously on 7 mg of *D*-Primordial(1-60)(A29C)-(HhH)$_2$. The progress of the reaction was followed by analytical HPLC (XBridge C4 column, 3.5 µm, 4.6 × 150 mm) with a gradient of 10-60% B over 20 min. *D*-Primordial-(HhH)$_2$, was purified by semi-preparative HPLC (XBridge BEH300 C4 column, 5 µm, 19 × 150 mm, method 25-50% B over 45 min) (**Figure S8**) to obtain 3.5 mg of *D*-Primordial-(HhH)$_2$ (50% yield).

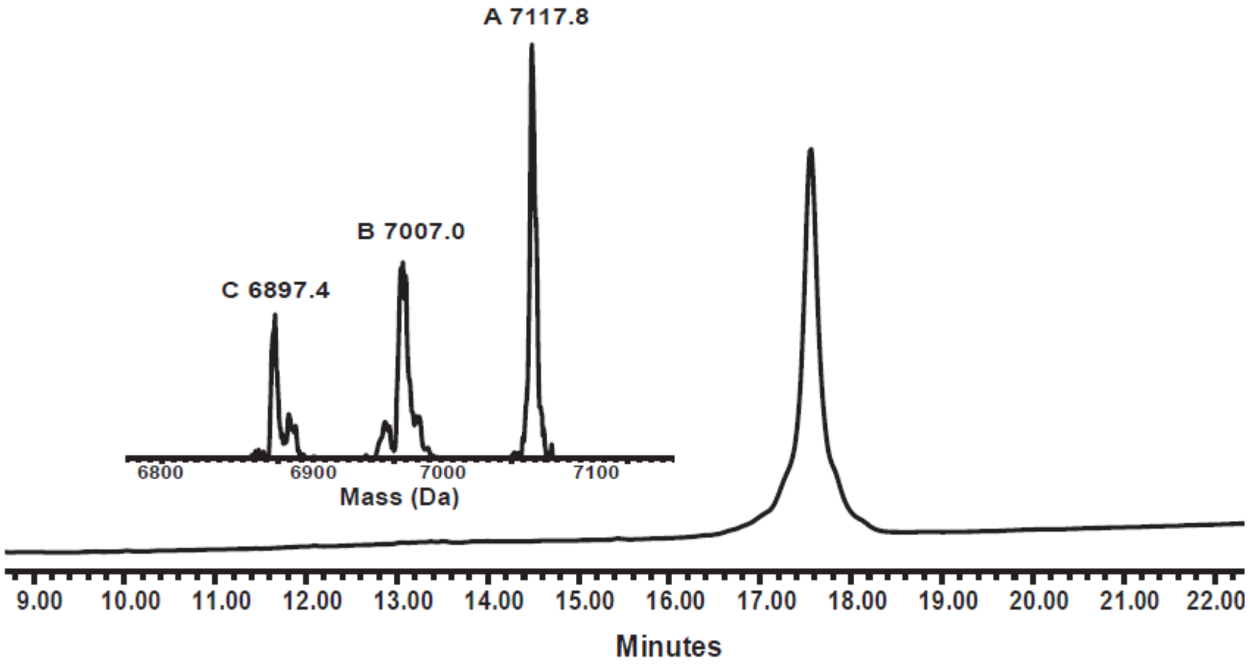

Figure S8. Desulfurization of *D*-Primordial(1-60)(A29C)-$(HhH)_2$. Analytical HPLC of the desulfurization reaction after 48 h with ESI-MS of desired product *D*-Primordial-$(HhH)_2$ (calc. 6896.1 Da; obs. 6897.4 Da, [M+TFA] 7007.0 Da, [M+ 2 TFA] 7117.8 Da).

Synthesis of *L*-Primordial-$(HhH)_2$-5G

Sequence:

RIRRASVEELTEV***GGGGG***RLARRILER**LA**SIERIRRASVEELTEV***GGGGG***RLARRILERL

The *L*-form of the disrupted Primordial protein was prepared from two peptide segments, NCL and desulfurization approach (**Scheme S2**). The sequence was similar to that of Primordial-$(HhH)_2$ except that the ***PGIGP*** binding motif was replaced by ***GGGGG*** (*bold and italics*). The two peptide segments were Primordial(1-28)-5G-$NHNH_2$, and Primordial(29-60)(A29C)-5G, in which Ala29 was temporary substituted with Cys to allow for the NCL reaction, and was later desulfurized to natural Ala29 after ligation. The ligation site is shown in *bold* and *underlined*. HPLC chromatograms and ESI-MS for purified peptide segments, ligation, and desulfurization products are presented in **Figures S9-S12**.

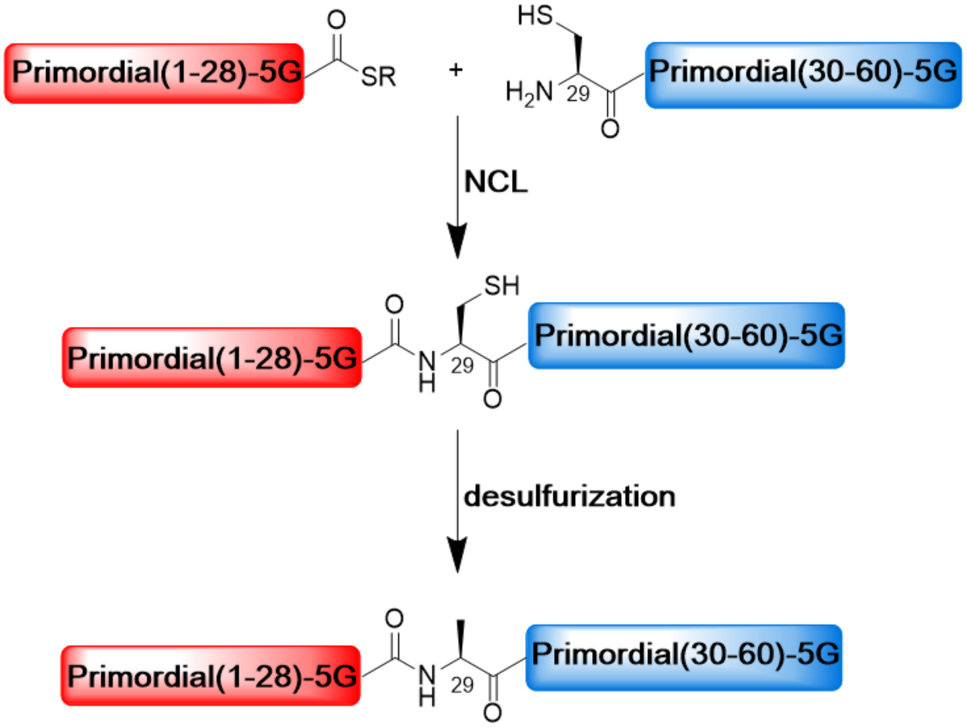

Scheme S2. **Synthesis of *L*-Primordial-(HhH)$_2$-5G**. Chemical protein synthesis scheme for *L*-Primordial-(HhH)$_2$-5G. The protein was prepared from two half-peptides and then joined using an NCL and desulfurization approach. The N-terminal half-peptide bears the C-terminal thioester, and the C-terminal peptide bears an N-terminal cysteine residue. After peptide ligation, the Cys residue is desulfurized to yield natural Ala.

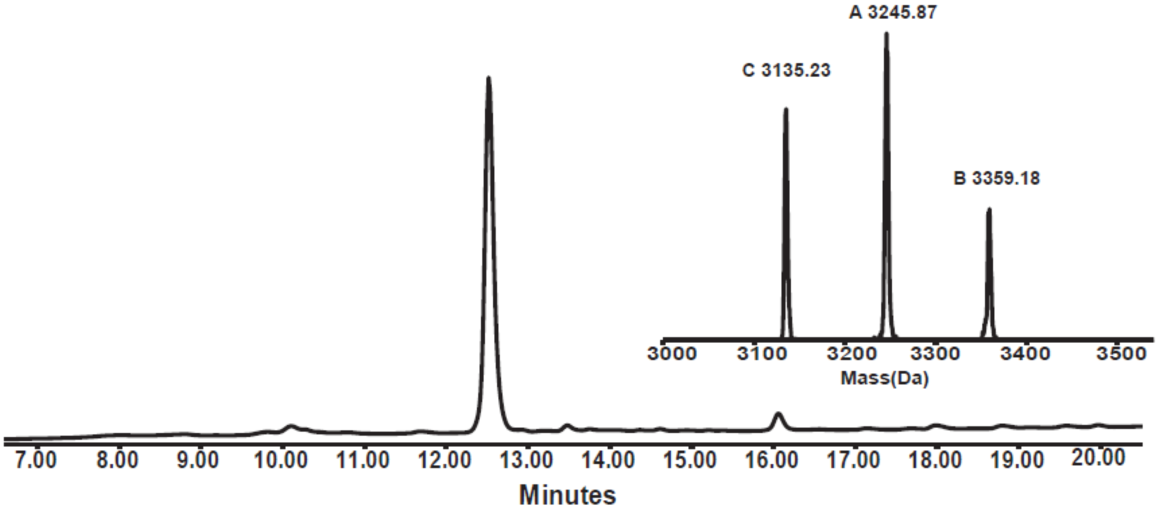

Figure S9. HPLC chromatograms and ESI-MS for purified *L*-Primordial(1-28)-5G-$NHNH_2$, with the inset showing the corresponding mass (calc. 3136.63 Da; obs. 3135.23 Da, [M+TFA] 3245.87 Da, [M+2 TFA] 3359.18 Da).

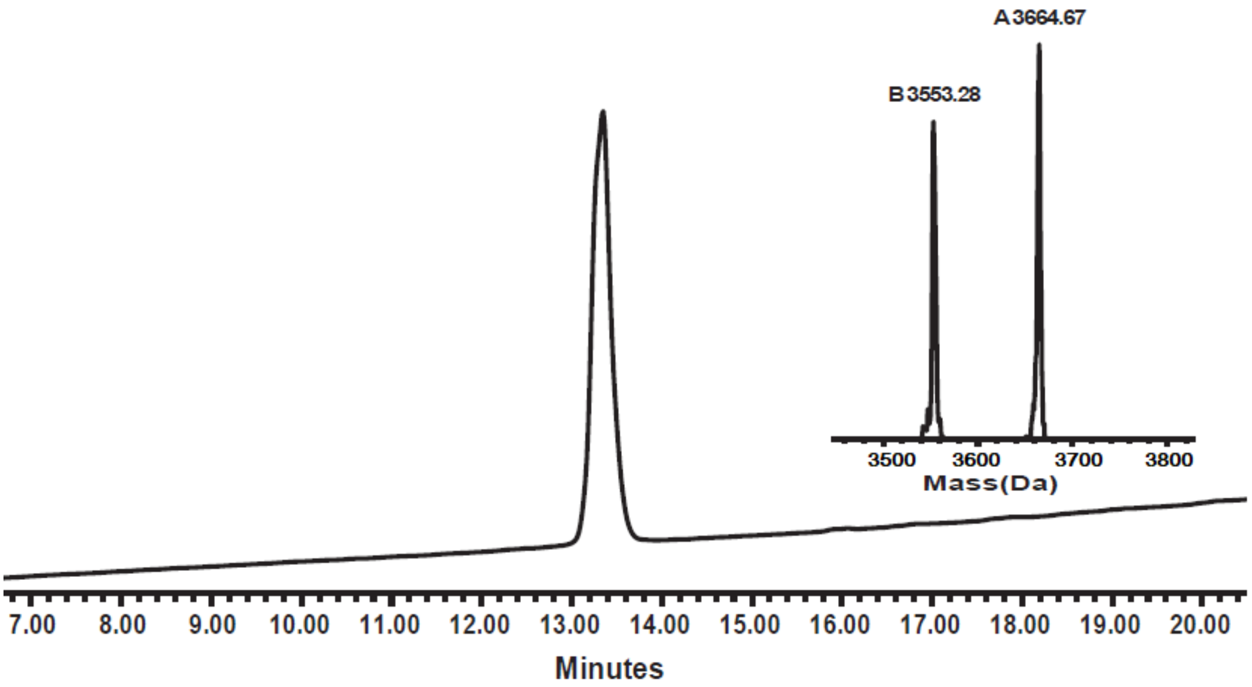

Figure S10. HPLC chromatograms and ESI-MS for purified *L*-Primordial(29-60)(A29C)-5G, with the inset showing the corresponding mass (calc. 3553.12 Da; obs. 3553.28 Da, [M+TFA] 3664.67 Da).

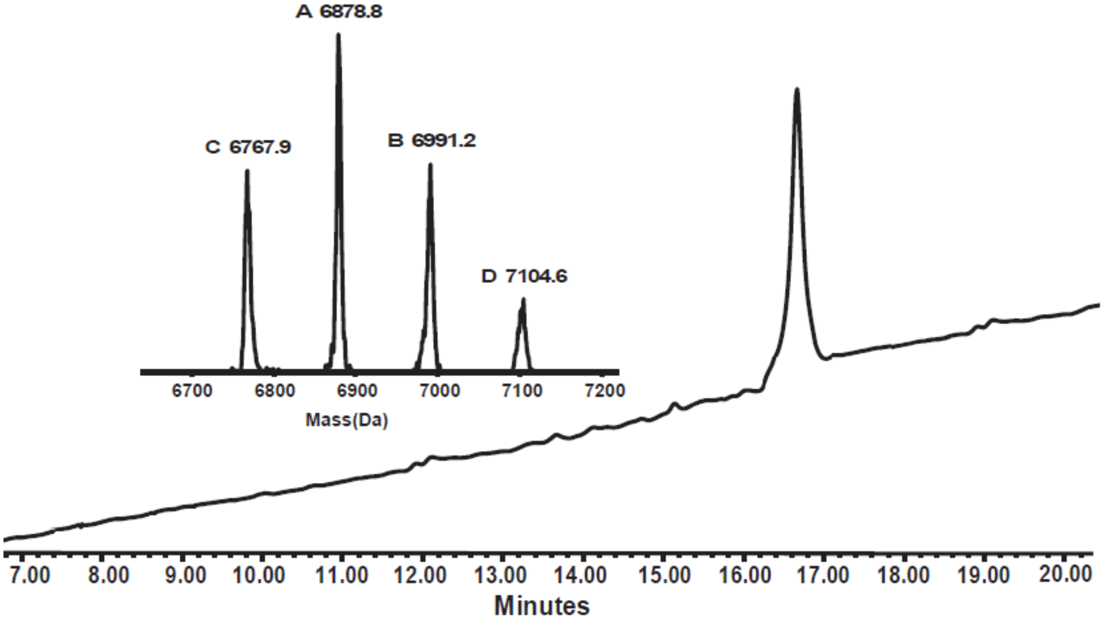

Figure S11. HPLC chromatograms and ESI-MS for *L*-Primordial-$(HhH)_2$(A29C)-5G with the inset showing the corresponding mass (calc. 6655.7 Da, [M+TFA] 6769.7 Da; obs. 6767.9 Da, [M+ 2 TFA] 6878.8 Da, [M+3 TFA] 6991.2 Da, [M+4 TFA] 7104.6 Da).

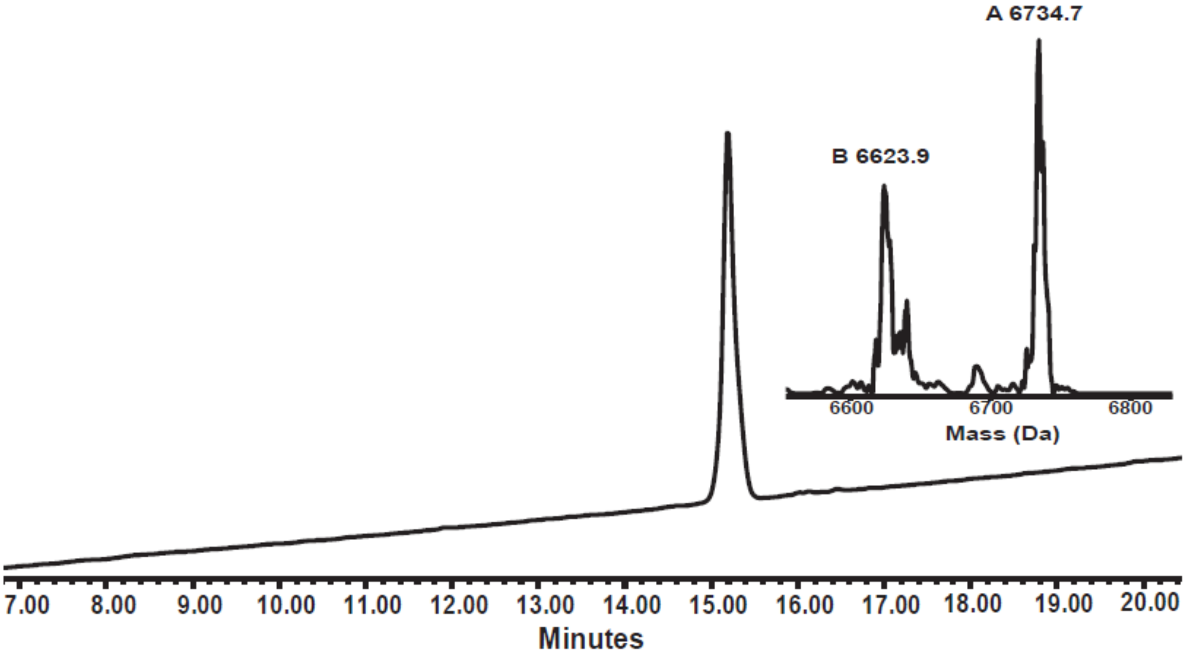

Figure S12. HPLC chromatograms and ESI-MS for purified *L*-Primordial-(HhH)$_2$-5G with the inset showing the corresponding mass (calc. 6623.7 Da; obs. 6623.9 Da, [M+ TFA] 6734.7 Da).

Synthesis of Ancestor-(HhH)$_2$

The total synthesis of Ancestor-(HhH)$_2$ is described in the **SI** of our previous article[9a].

Synthesis of *D/L*-Precursor-HhH

Sequence:
rIrRaSvEeLtEvPGIGPrLaRrIlErLa

Single HhH peptides in which *D*- and *L*-amino acids were coupled alternatively (*D*-amino acids are in small letters and underlined in the sequence shown above) were synthesized by an automatic peptide synthesizer (CS136XT, CS Bio Inc. CA) on a 0.15 mmol Rink amide resin (RAPP Polymer, loading 0.19) as described above. Arg residues were doubly coupled and all the *D*-amino acids were manually coupled. The peptide *D/L*-Precursor-HhH was then cleaved as described above yielding 264 mg of crude peptide.

Purification: The peptide was purified by RP-HPLC (50 mg of crude peptide) on an XSelect C4 column (5 µm, 130 Å, 19× 250 mm) using a gradient of 25-45% B over 42 min to give pure *D/L*-

Precursor-HhH (8 mg, 16% yield). The HPLC analysis (**Figure S13**) was carried out on a C4 analytical column.

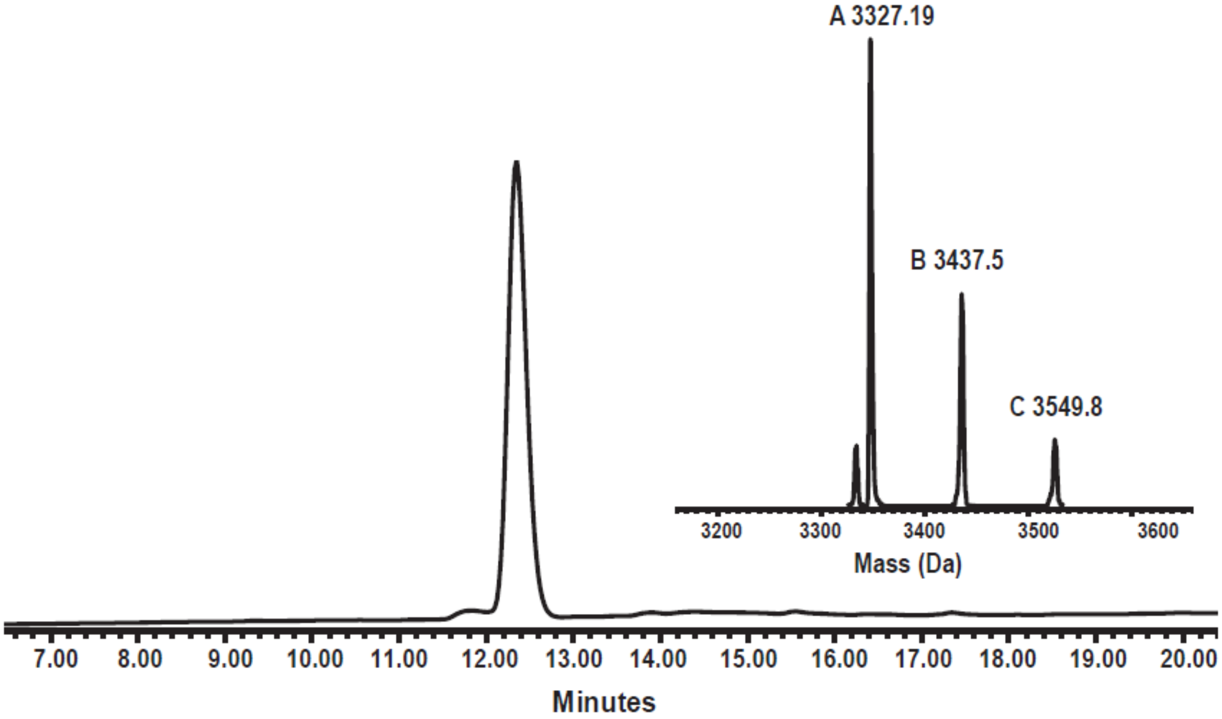

Figure S13. HPLC chromatograms and ESI-MS for purified *D/L*-Precursor-HhH with the inset showing the corresponding mass. (calc. 3326.96 Da; obs. 3327.19 Da, [M+TFA] 3437,5 Da, [M+2 TFA] 3549.8 Da).

Proteins labeled with Cy5-NHS Ester for MST studies

L-Primordial-$(HhH)_2$ and *L*-Primordial-$(HhH)_2$-5G were labeled with Cy5-NHS Ester for measurement with Microscale Thermophoresis (MST). For the labeling reaction, a stock solution of labeling reagent was prepared by dissolving 1 mg of Cy5-NHS ester in 200 µL DMF (8.12 mM). Then, 1 mg of protein was dissolved in 0.5 mL 0.2 M PBS pH 8.5 (conc. ~0.29 mM), and 71 µL of the Cy5-NHS ester stock solution (4 equiv, conc. ~1.16 mM) were added to the protein and vortexed well. The reaction was kept at room temperature for approximately 6 hours [https://www.lumiprobe.com/protocols/nhs-ester-labeling]. The reaction was monitored by HPLC and ESI-MS. Labeled peptides were purified by an XSelect CSH C18 column (5 µm, 130 Å, 10 × 150 mm) using a gradient of 30-50% B over 55 min. The labeled peptides were further analyzed using HPLC on a C4 analytical column (**Figures S14-S15**). Yields of the labeling reactions were 70-85% after the purification step.

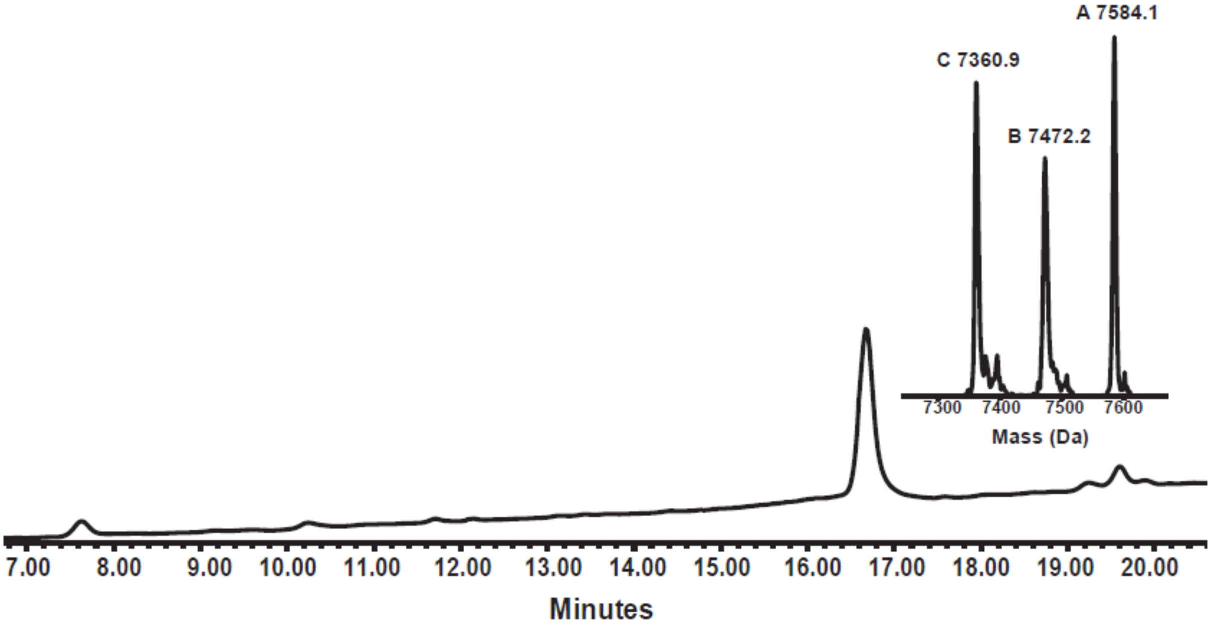

Figure S14. HPLC chromatograms and ESI-MS for purified *L*-Primordial-$(HhH)_2$ labeled with Cy5, with the inset showing the corresponding mass (calc. 7361.8 Da; obs. 7360.9 Da, [M+TFA] 7472.2 Da, [M+2 TFA] 7584.1 Da).

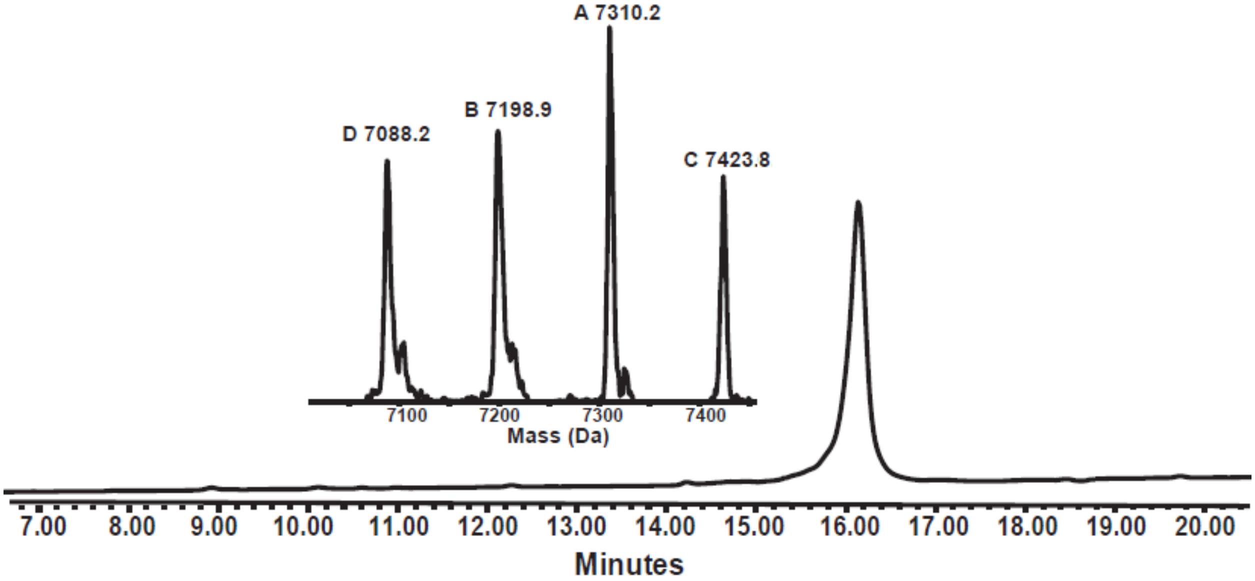

Figure S15. HPLC chromatograms and ESI-MS for purified *L*-Primordial-$(HhH)_2$-5G labeled with Cy5, with the inset showing the corresponding mass. (calc. 7089.3 Da; obs. 7088.2 Da, [M+1 TFA] 7198.9 Da] [M+2 TFA] 7310.2 Da, [M+3 TFA] 7423.8 Da).

High-Resolution Mass Spectrometry of Proteins and Peptides Synthesized in this Work

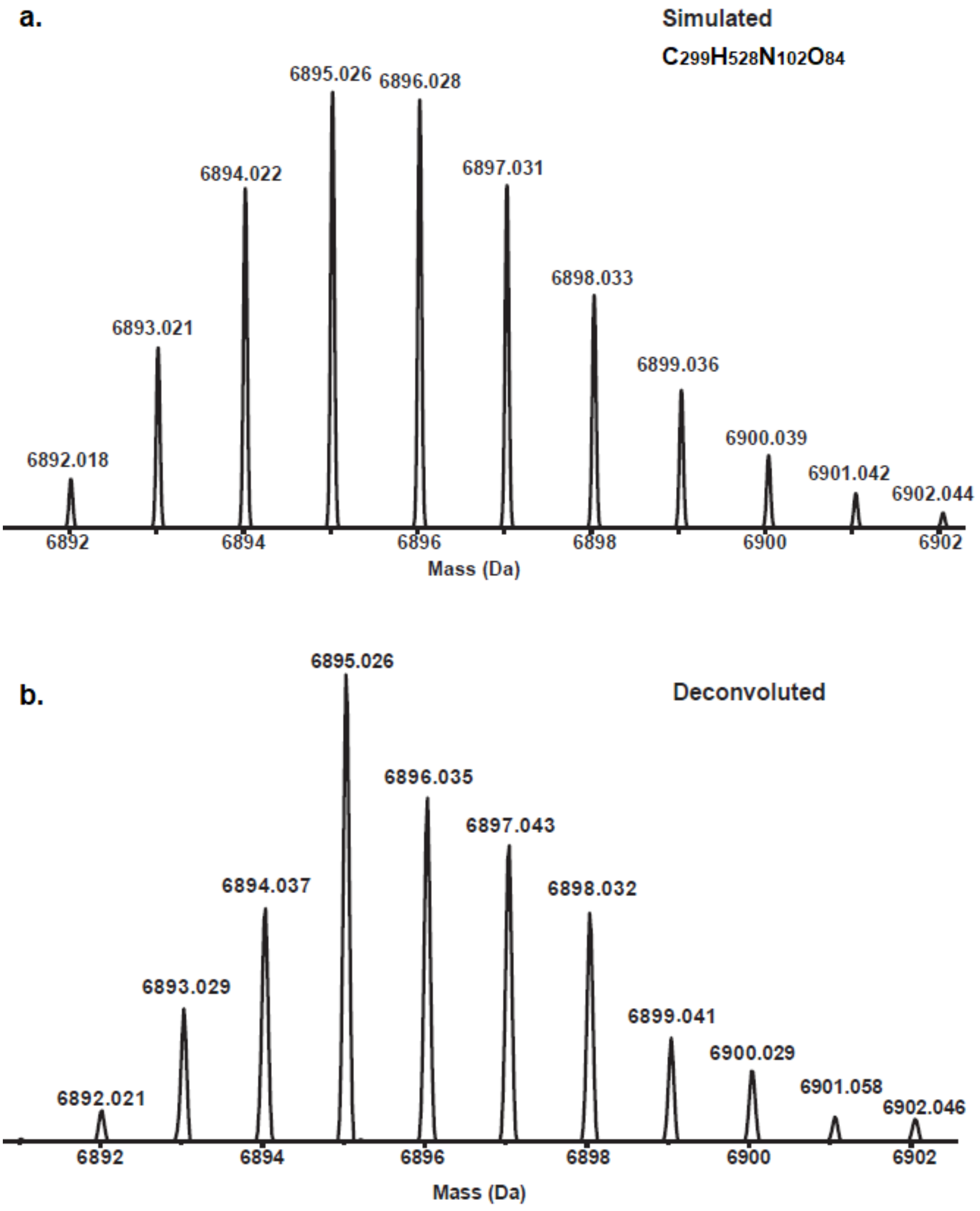

Figure S16. HR-MS analysis of *L*-Primordial-$(HhH)_2$. **a**. The simulated spectrum with chemical formula $C_{299}H_{528}N_{102}O_{84}$ is shown; **b**. The deconvoluted spectrum.

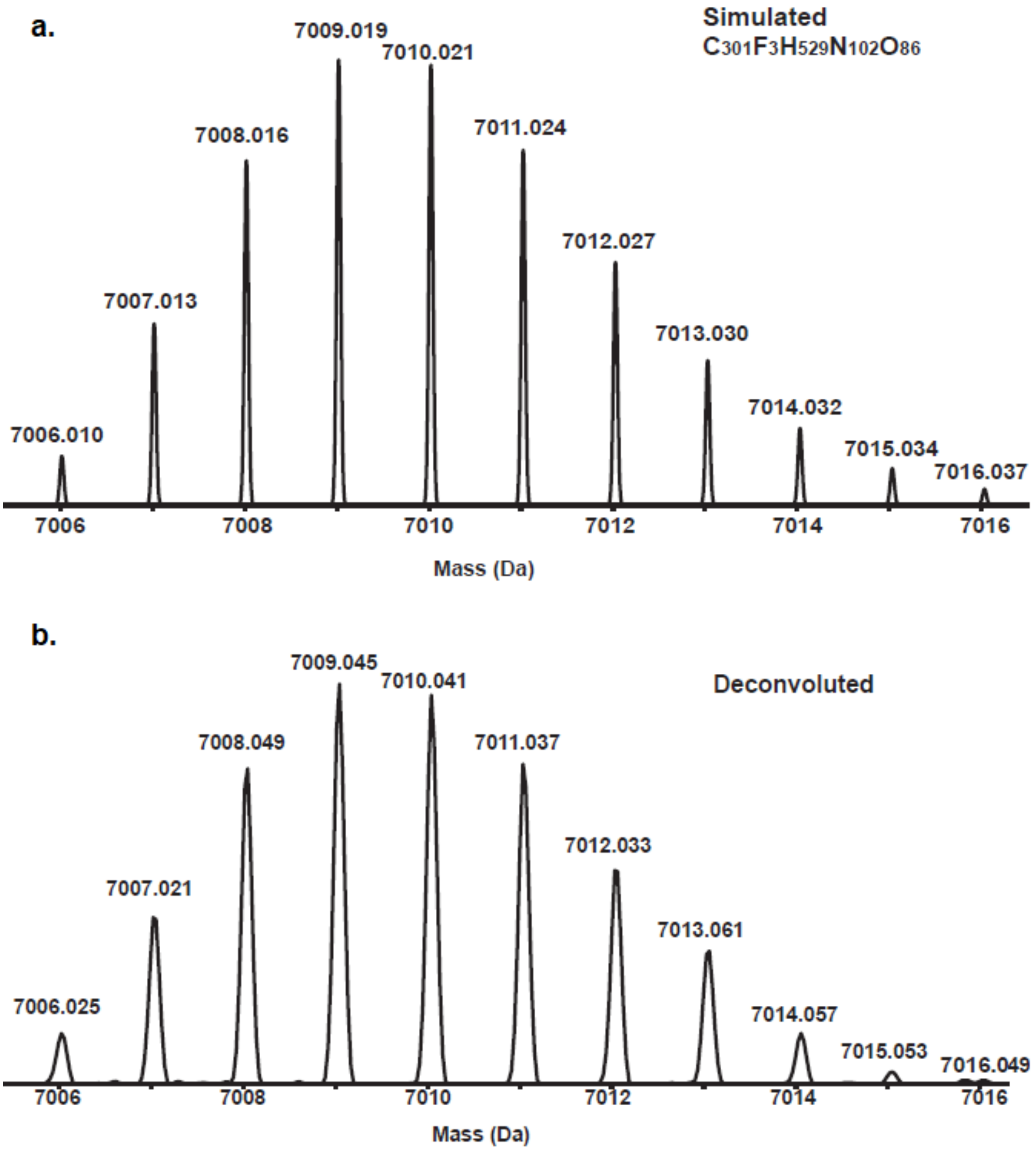

Figure S17. HR-MS analysis of *D*-Primordial-$(HhH)_2$. **a**. The simulated spectrum with one TFA molecule adduct, chemical formula $C_{301}F_3H_{529}N_{102}O_{86}$ is shown; **b**. The deconvoluted spectrum.

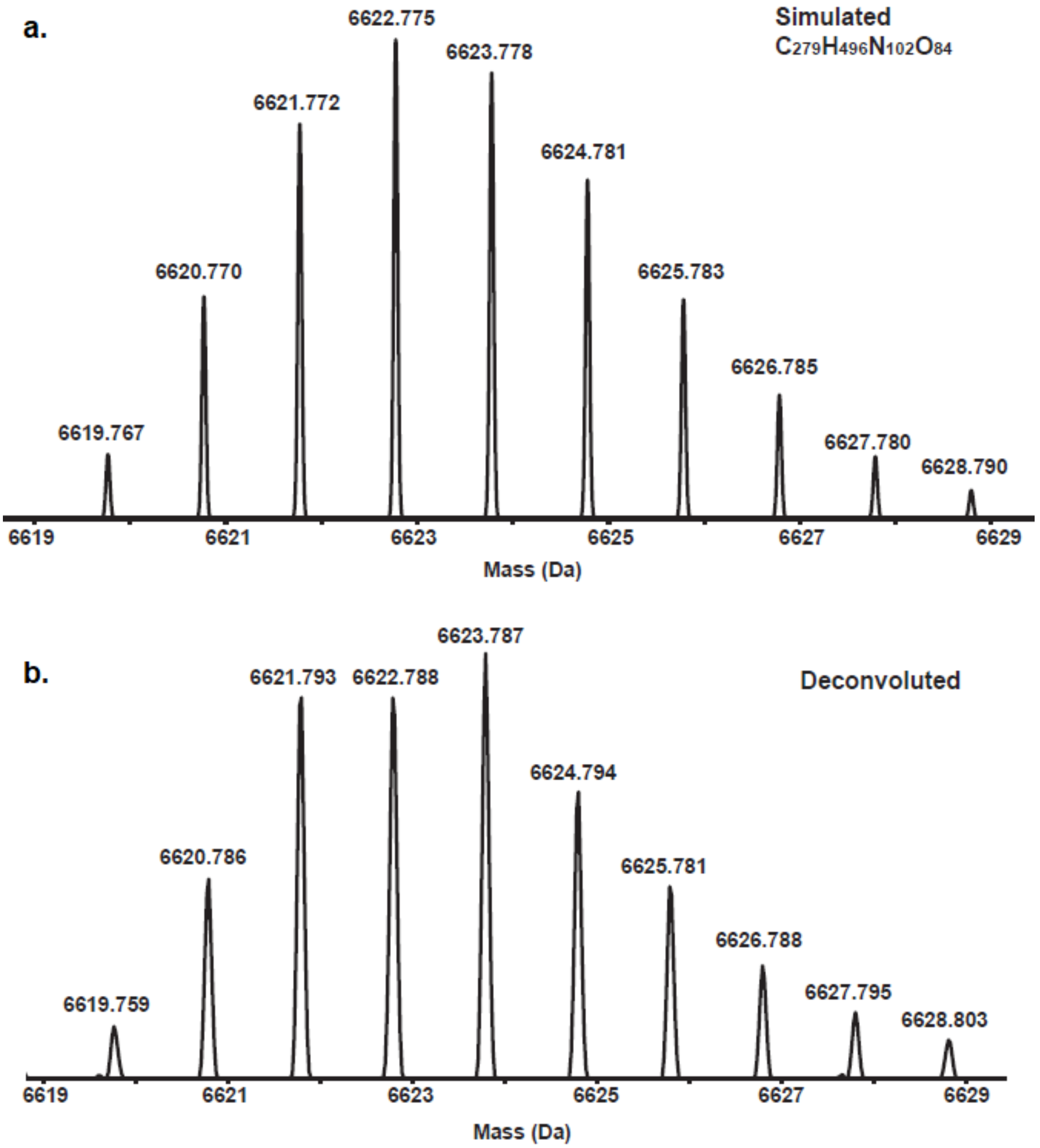

Figure S18. HR-MS analysis of *L*-Primordial-$(HhH)_2$-5G. **a**. The simulated spectrum with chemical formula $C_{279}H_{496}N_{102}O_{84}$ is shown; **b**. The deconvoluted spectrum.

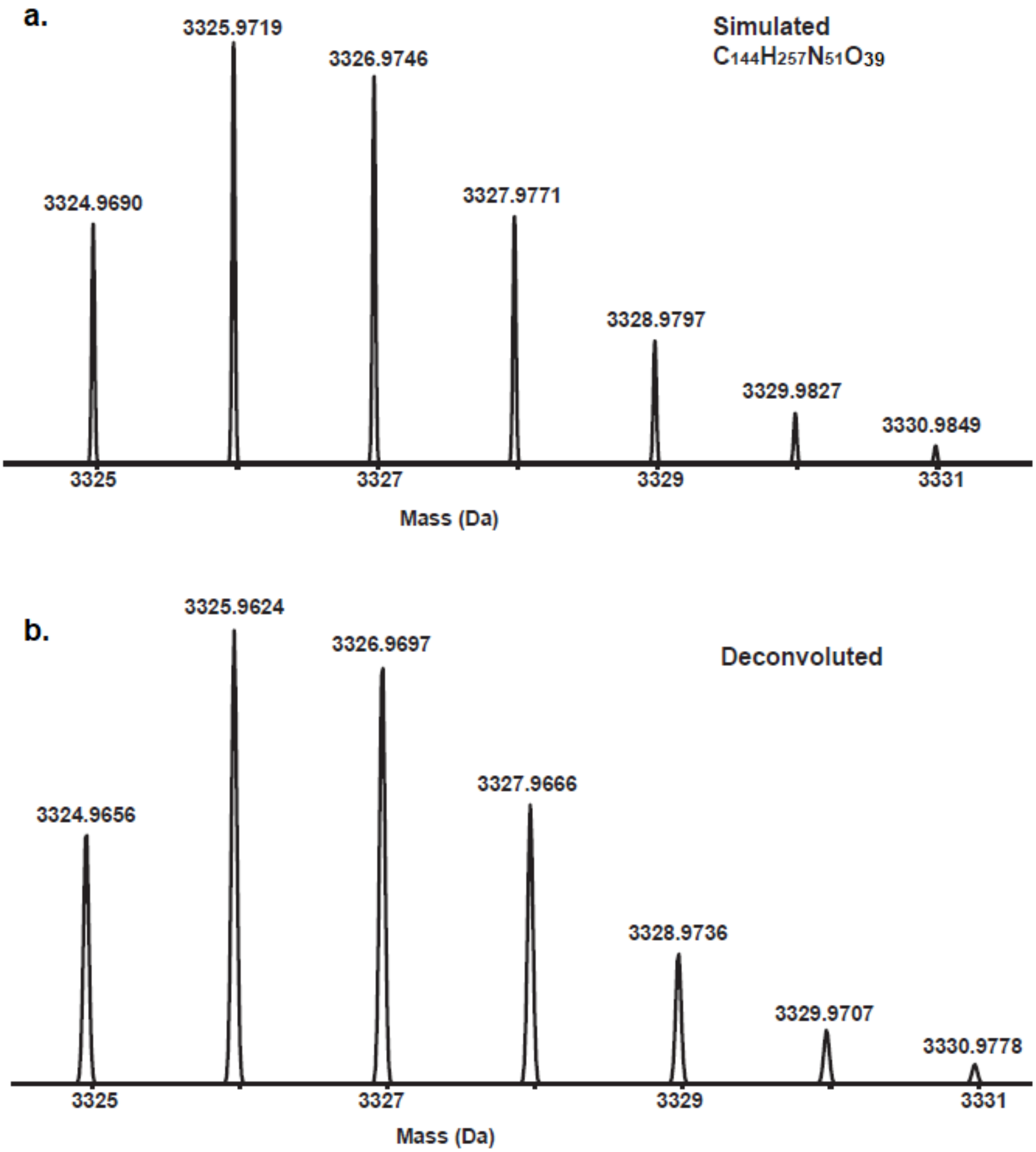

Figure S19. HR-MS analysis of *D/L*-Precursor-HhH. **a**. The simulated spectrum with chemical formula $C_{144}H_{257}N_{51}O_{39}$ is shown; **b**. The deconvoluted spectrum.

Structural alignment

Metal type	AF3	Mean ipTM	Mean peptide pTM	GIG motifs
None	No binding	0.13	0.75	2087 (7)
NA	Binding	0.35	0.83	1310 (12)
MG	Binding	0.3	0.82	354 (3)
CA	Binding	0.43	0.83	207 (2)
K	Binding	0.33	0.82	49
ZN	Binding?	0.43	0.81	0
MN	-	-	-	8
NI	-	-	-	3
BA	-	-	-	2
CS	-	-	-	2
CR	-	-	-	1
			Total:	4023

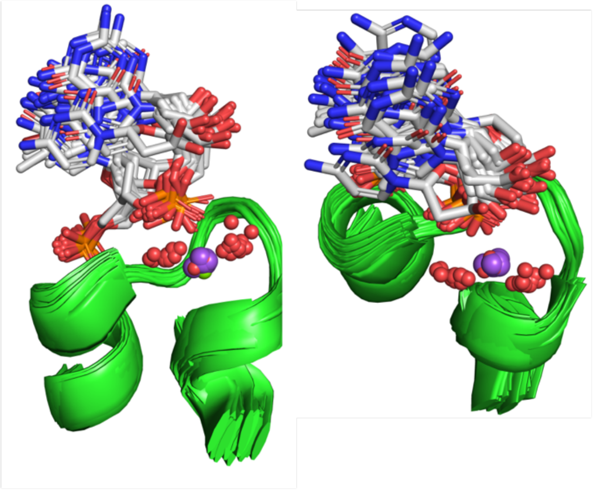

Figure S20. Types of metal atoms bound by the HhH hairpin motifs. AlphaFold3 predicts $(HhH)_2$-fold binding to dsDNA relies on a bridging cation, either Na^+, Mg^{2+}, Ca^{2+}, or K^+ ions (left table, *gray* area). Although Zn^{2+} ions also allowed the $(HhH)_2$-Fold to bind dsDNA in the expected binding mode, the Zn^{2+} metals did not occupy the conserved metal position. Mean ipTM (protein + dsDNA + ions) and pTM (protein) were calculated from 5 structures produced by AlphaFold3[13]. Although mean iPTM scores are generally low, the binding mode correctly predicts the location of the conserved metal based on available crystal structures. To analyze the patterns of metal ion utilization in the canonical HhH binding mode, 7,562 domains from the ECOD database (version 291)[14] containing X-group 102 ('HhH/H2TH') were clustered by T-group and aligned using MAFFT L-INS-i[15] to identify the nucleic acid-binding motif positions. PDB structures with a nucleic acid within 3.5 Å of the binding motif G*h*G (where *h* is a hydrophobic residue) were identified. In total, 4,023 nucleic acid-binding HhH motifs were detected, corresponding to 3,663 sequences. For structural alignment (center: side view, right: top view), representative sequences were chosen from each of the 531 CD-HIT clusters[16] (sequence identity cutoff = 0.9) based on resolution (≤ 2.5 Å) and the presence of nucleic acids. 46 structures met the above criteria, from which 23 sequences with 24 motifs (with one of the sequences containing 2 motifs) were chosen as representatives. Representative structures span 7 ECOD F-groups. Waters are indicated with *red* spheres, sodium with *purple* spheres, and magnesium/calcium with *green* spheres. Although not clearly visible, 6 representative structures (e3cwsA1, e5yuyA1, e6jumA1, e6cstA3, e7k00M1, and e3f2bA5) model a water molecule in place of the conserved metal ion, likely in error. The ECOD domains used in this alignment are as follows: e1ornA1, e3s6iA1, e4ejyA3, e3i0wA1, e4i2aA2, e4kliA1, e4kliA2, e3osnA1, e4p4mA1, e2ihmA4, e4p4oA3, e2bcqA1, e2xhiA3, e6x6zA2, e4ofaA1, e3gqcA2, e4y4o1m1, e3cwsA1, e5yuyA1, e6jumA1, e6cstA3, e7k00M1, e3f2bA5, and e3v7kA1. Together these structures span ECOD F-groups 102.1.1.21, 102.1.1.23, 102.1.1.28, 102.1.1.30, 102.1.1.51, 102.1.2.1, and 102.2.1.2.

Construct Characterization

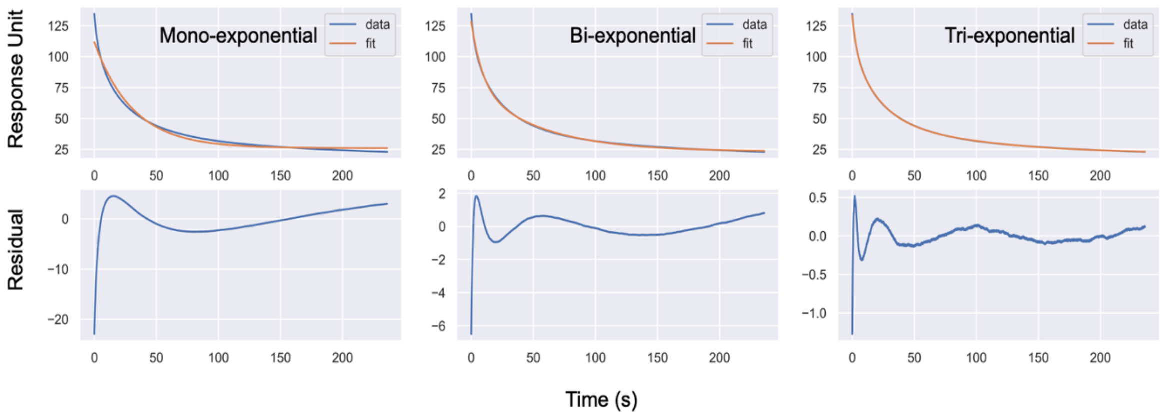

Figure S21. Representative dissociation curves. Within the window of useful SPR data, three kinetic phases can be reliably fitted. Although additional minor phases may be present, they could not be reliably fitted.

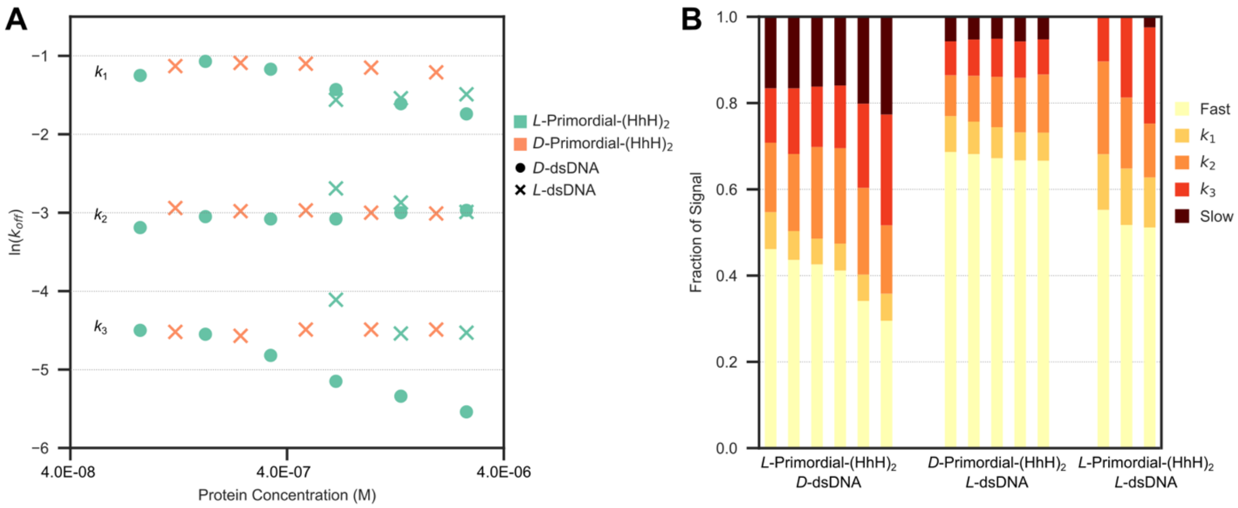

Figure S22. Analysis of SPR dissociation kinetics. **A.** Kinetic phases associated with dissociation from dsDNA. As expected, off rates are largely concentration independent. Note that the dissociation rate constants associated with *L*-Primordial-$(HhH)_2$ binding to *D*-dsDNA is similar to the mirror image pair (*D*-Primordial-$(HhH)_2$ dissociating from *L*-dsDNA) and, surprisingly, also similar to the dissociation of *L*-Primordial-$(HhH)_2$ from *L*-dsDNA. Fit errors are smaller than the datapoints. **B**. The fraction of signal associated with each kinetic phase. Note that dissociation kinetics of the natural chiral pair are complex, and are associated with at least 5 kinetic phases: A fast phase (or phases) that occurs during a period of mechanical noise (due to SPR needle movements) and high error (due to a refractive index change of the buffer); three kinetic phases that can be accurately modeled (see **Figure S21**); and a slow phase (or phases) that manifests as a positive shift in the baseline (and residual signal at $t = \infty$) but that can be eluted with 2M NaCl. The three phases that can be accurately modeled are relatively concentration independent. However, at higher concentrations of *L*-Primordial-$(HhH)_2$, the slowest of these three kinetic processes becomes slightly slower. This change may be due to cooperative binding effects as the dsDNA becomes progressively more coated with protein molecules that can then start interacting with each other and/or stabilize the optimal conformation of the dsDNA for binding. The flux through each kinetic mode associated with the mirror image pair are highly similar to the natural pair, as expected, though with a notably higher contribution of the fast phase and a lower contribution of the slow phase. This difference is likely due to the lower synthetic purity of the *D*-protein and the *L*-RNA. Remarkably, the three measurable kinetic phases associated with *L*-protein binding to *D*-dsDNA are retained in *L*-protein binding to *L*-dsDNA. This conservation of kinetic phases may suggest a conservation of some binding modes. However, in the case of *L*-protein binding to *L*-dsDNA, the slow kinetic phase was almost completely abolished, perhaps suggesting the loss of the most stable binding mode.

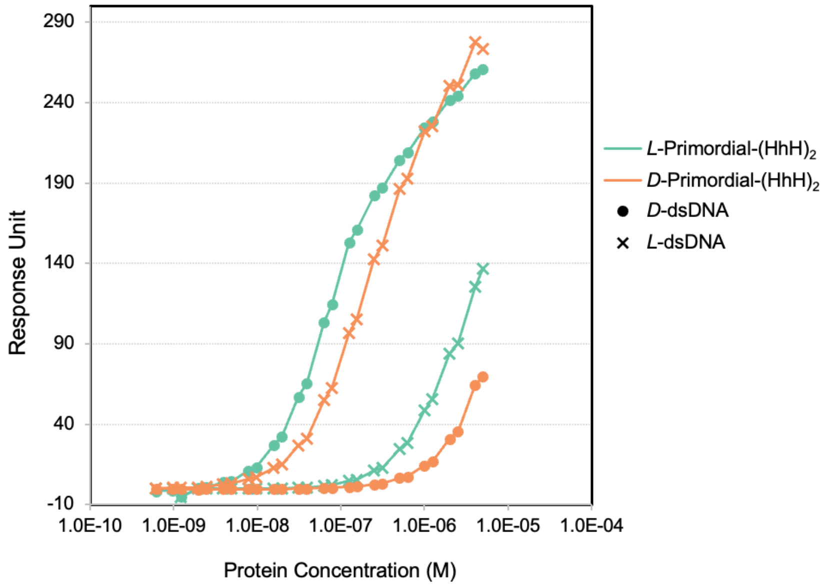

Figure S23. SPR steady state analysis of *L*- and *D*-Primordial-$(HhH)_2$ binding to *D*- and *L*-dsDNA. Note that these data were collected on a different SPR chip than that presented in **Figure 3** of the **Main Text**. As in **Figure 3**, steady state binding was estimated after 216 seconds of injection.

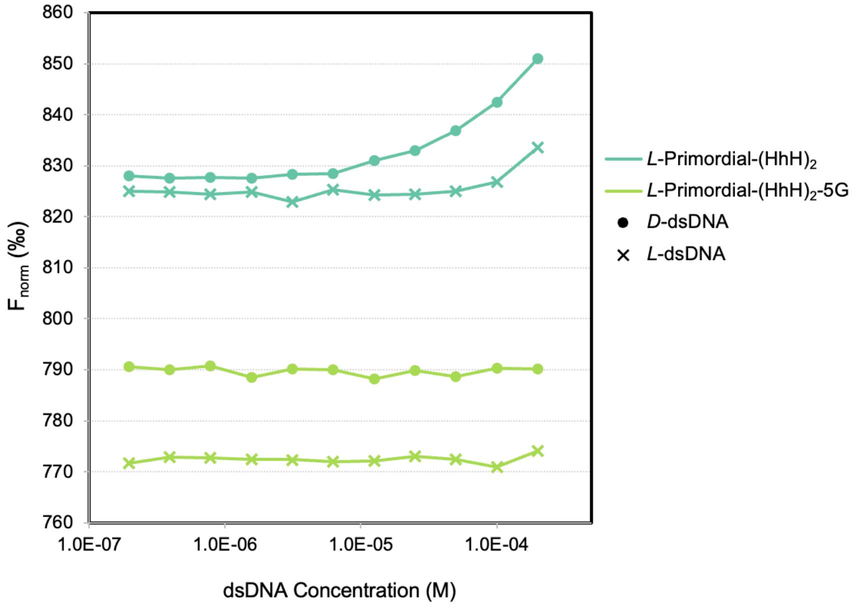

Figure S24. Microscale thermophoresis analysis. *L*-Primordial-$(HhH)_2$-5G, which has a disrupted PGIGP motif, does not bind to dsDNA of either chirality. *L*-Primordial-$(HhH)_2$, on the other hand, has indications of binding for both *L*- and *D*-dsDNA. Unfortunately, higher concentrations of ligands could not be analyzed due to significant changes in initial fluorescence of the conjugated Cy5 dye.

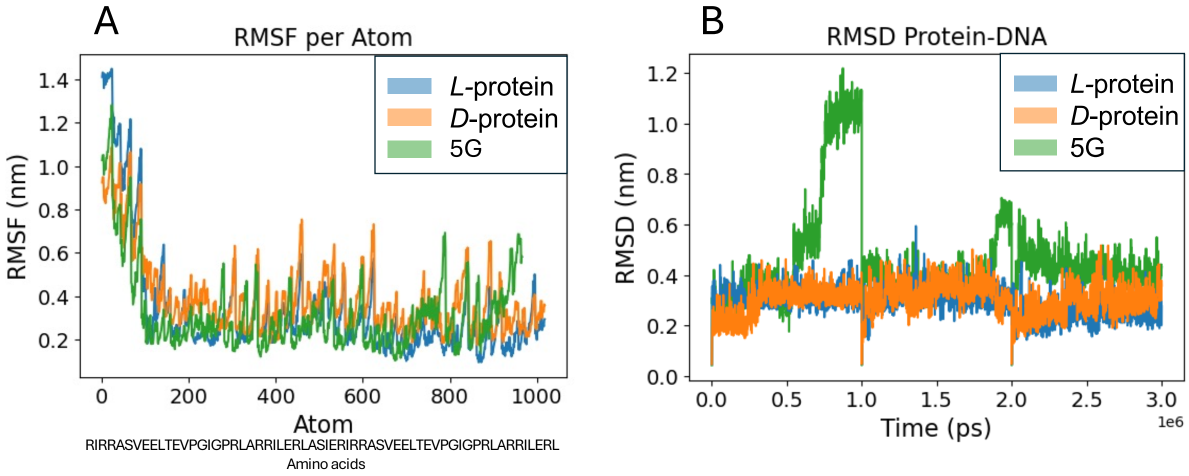

Figure S25. **Structural fluctuations and RMSD distribution of protein-dsDNA complexes**. **A**. RMSF *per* atom for *L*-Primordial-(HhH)$_2$ (*L*-protein, *blue*), *D*-Primordial-(HhH)$_2$ (*D*-protein, *orange*), and *L*-Primordial-(HhH)$_2$-5G control (*green*) systems. All systems show low residue flexibility, with fluctuations observed only in the first seven N-terminal amino acids (atoms 1-120), indicating that the HhH motifs remain stable. **B**. RMSD over time for the *L*-Primordial-(HhH)$_2$ (*blue*), *D*-Primordial-(HhH)$_2$ (*orange*), and *L*-Primordial-(HhH)$_2$-5G (*green*) systems. Both *L*- and *D*-proteins show stable binding with low RMSD values, while the 5G control fluctuates, indicating weak or no binding. Each of the three simulations are represented in the same plot as a continuous time series.

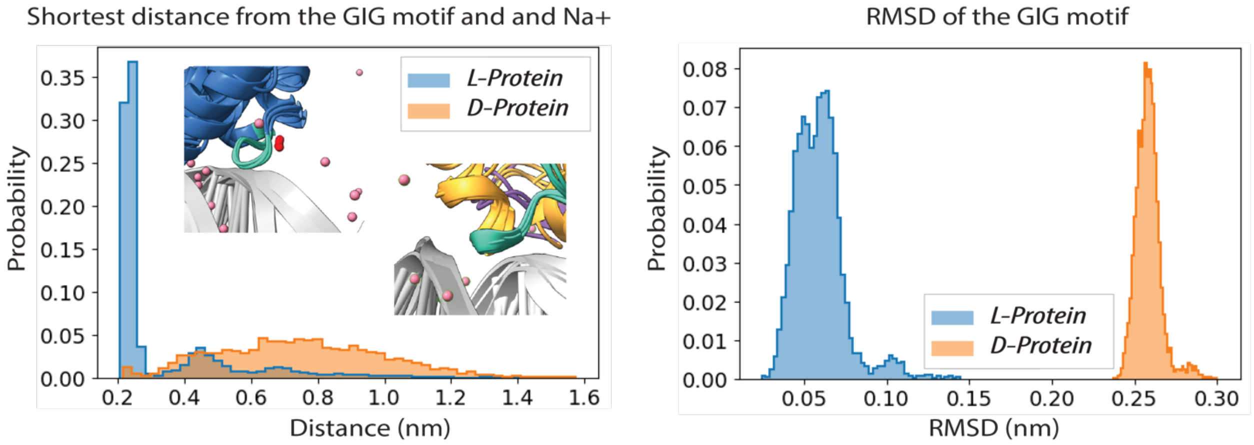

Figure S26. **Structural analysis of the PGIGP motif in *L and D*-proteins: Left,** Histogram of the shortest distances between PGIGP motifs and the closest sodium ion. **Right,** Histogram of RMSD values for the PGIGP motifs in *L*-protein (*blue*) and *D*-protein (*orange*). The sodium ions that are close to the PGIGP motif are shown as *red* spheres and all other sodium ions are shown in *pink*. It is evident that no sodium ion is close to the PGIGP motif in the *D*-protein.

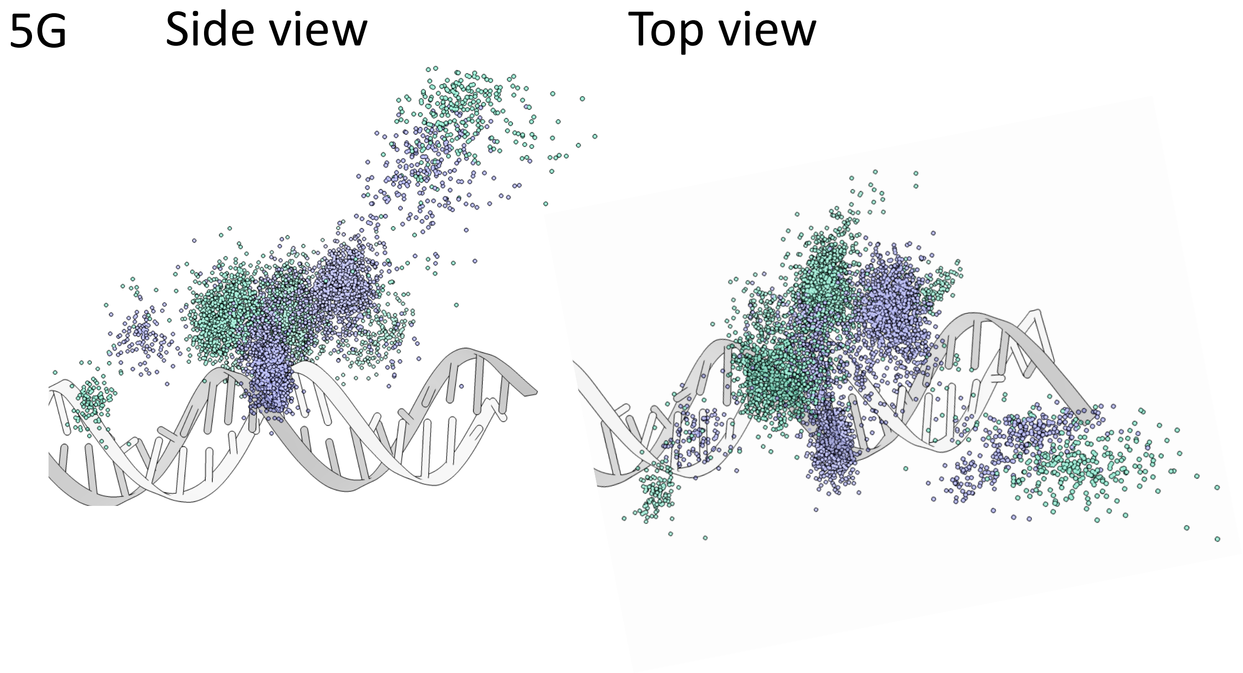

Figure S27. ***L*-Primordial-(HhH)$_2$-5G motif distributions**. The side and top views show the center of mass (COM) distributions for the binding motifs (canonically PGIGP, but mutated to GGGGG here) in the *L*-Primordial-(HhH)$_2$-5G system. The binding motifs are represented in *cyan* and *purple*, corresponding to the two PGIGP regions within the protein (which are mutated to GGGGG in the 5G construct). Unlike the compact localization observed in the *L*- and *D*-protein systems, the motifs in the 5G system are widely scattered across the DNA surface. This scattered distribution confirms the absence of stable and specific interactions with the DNA, highlighting the destabilized nature of the 5G variant.

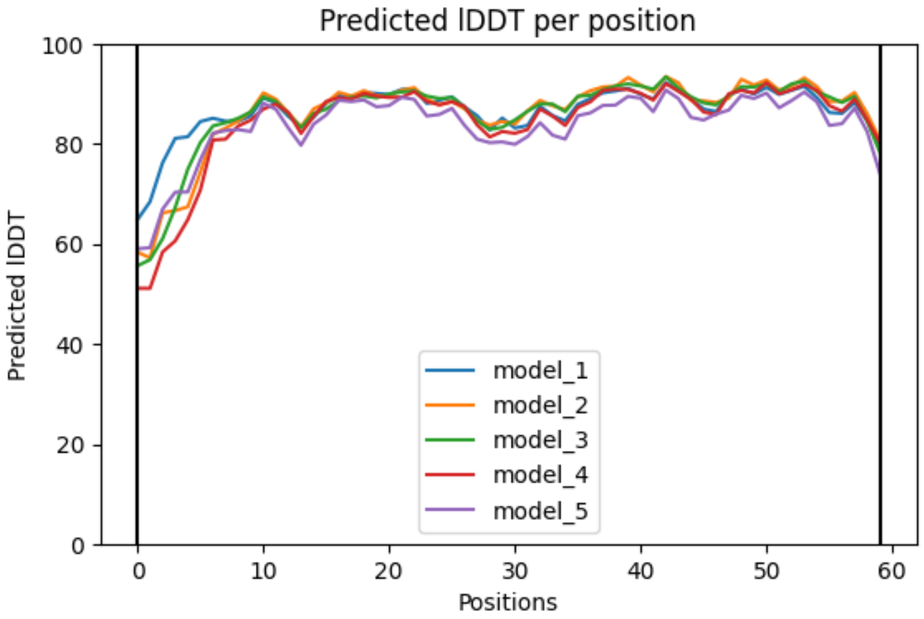

Figure S28. plDDT scores of *L*-Primordial-(HhH)$_2$ in the absence of DNA predicted by AlphaFold2. Scores above 90 indicate high accuracy, 70–90 suggest reliable backbone placement with minor side-chain uncertainty, and values below 50 point to low-confidence regions, often representing flexible or disordered regions. The N-terminus, which is also the most disordered in MD simulations (**Figure S25**) has the lowest prediction accuracy.

Table S1. DNA sequences.

Experiment	Sequence
SPR and MST<br>*D*- and *L*-dsDNA<br>(**Figure 3C-E, S23, S24**)	5’biotin-CCGTCCGTAATCATGGTCATAGCTGTTTC-3'<br>(Reverse complement strand not biotinylated)
MD<br>*D*-dsDNA<br>(**Figure 4, S25**)	5’-CGCTAGATCGATCGCTAGATC-3’

References

[1] J. Schindelin, I. Arganda-Carreras, E. Frise, V. Kaynig, M. Longair, T. Pietzsch, S. Preibisch, C. Rueden, S. Saalfeld, B. Schmid, J. Y. Tinevez, D. J. White, V. Hartenstein, K. Eliceiri, P. Tomancak, A. Cardona, *Nat Methods* **2012**, *9*, 676-682.

[2] D. Van Der Spoel, E. Lindahl, B. Hess, G. Groenhof, A. E. Mark, H. J. Berendsen, *J Comput Chem* **2005**, *26*, 1701-1718.

[3] O. Guvench, S. S. Mallajosyula, E. P. Raman, E. Hatcher, K. Vanommeslaeghe, T. J. Foster, F. W. Jamison, 2nd, A. D. Mackerell, Jr., *J Chem Theory Comput* **2011**, *7*, 3162-3180.

[4] J. A. Lemkul, *Living Journal of Computational Molecular Science* **2018**, *1*, 5068.

[5] A. J. Malik, P. G. A. Aronica, C. S. Verma, *Structure* **2020**, *28*, 1376-1378.

[6] R. B. Merrifield, *J Am Chem Soc* **1963**, *85*, 2149-2154.

[7] P. E. Dawson, T. W. Muir, I. Clark-Lewis, S. B. H. Kent, *Science* **1994**, *266*, 776-779.

[8] a) L. Z. Yan, P. E. Dawson, *J Am Chem Soc* **2001**, *123*, 526-533; b) Q. Wan, S. J. Danishefsky, *Angew Chem Int Ed* **2007**, *46*, 9248-9252.

[9] a) L. M. Longo, D. Despotovic, O. Weil-Ktorza, M. J. Walker, J. Jablonska, Y. Fridmann-Sirkis, G. Varani, N. Metanis, D. S. Tawfik, *Proc Natl Acad Sci U S A* **2020**, *117*, 15731-15739; b) M. Seal, O. Weil-Ktorza, D. Despotović, D. S. Tawfik, Y. Levy, N. Metanis, L. M. Longo, D. Goldfarb, *J Am Chem Soc* **2022**, *144*, 14150-14160.

[10] J. B. Blanco-Canosa, P. E. Dawson, *Angew. Chem. Int. Ed.* **2008**, *47*, 6851-6855.

[11] J. S. Zheng, S. Tang, Y. K. Qi, Z. P. Wang, L. Liu, *Nat. Protoc.* **2013**, *8*, 2483-2495.

[12] D. T. Flood, J. C. J. Hintzen, M. J. Bird, P. A. Cistrone, J. S. Chen, P. E. Dawson, *Angew Chem Int Edit* **2018**, *57*, 11634-11639.

[13] J. Abramson, J. Adler, J. Dunger, R. Evans, T. Green, A. Pritzel, O. Ronneberger, L. Willmore, A. J. Ballard, J. Bambrick, S. W. Bodenstein, D. A. Evans, C. C. Hung, M. O'Neill, D. Reiman, K. Tunyasuvunakool, Z. Wu, A. Zemgulyte, E. Arvaniti, C. Beattie, O. Bertolli, A. Bridgland, A. Cherepanov, M. Congreve, A. I. Cowen-Rivers, A. Cowie, M. Figurnov, F. B. Fuchs, H. Gladman, R. Jain, Y. A. Khan, C. M. R. Low, K. Perlin, A. Potapenko, P. Savy, S. Singh, A. Stecula, A. Thillaisundaram, C. Tong, S. Yakneen, E. D. Zhong, M. Zielinski, A. Zidek, V. Bapst, P. Kohli, M. Jaderberg, D. Hassabis, J. M. Jumper, *Nature* **2024**, *636*, E4.

[14] H. Cheng, R. D. Schaeffer, Y. Liao, L. N. Kinch, J. Pei, S. Shi, B. H. Kim, N. V. Grishin, *PLoS Comput Biol* **2014**, *10*, e1003926.

[15] K. Katoh, D. M. Standley, *Mol Biol Evol* **2013**, *30*, 772-780.
[16] W. Li, A. Godzik, *Bioinformatics* **2006**, *22*, 1658-1659.
